# Temporal Patterns of Allergen Sensitisation in the General Population: The LEAD Study

**DOI:** 10.1002/clt2.70181

**Published:** 2026-06-12

**Authors:** Charmaine J. M. Lim, Abdelrahman Omar, Severin Pötscher, Marie‐Kathrin Breyer, Frits M. E. Franssen, Emiel F. M. Wouters, Robab Breyer‐Kohansal

**Affiliations:** ^1^ Ludwig Boltzmann Institute for Lung Health Vienna Austria; ^2^ Sigmund Freud Private University Faculty of Medicine Vienna Austria; ^3^ Department of Respiratory and Pulmonary Diseases Clinic Hietzing Vienna Healthcare Group Vienna Austria; ^4^ Department of Respiratory and Pulmonary Diseases Site Penzing of Clinic Ottakring Vienna Healthcare Group Vienna Austria; ^5^ NUTRIM Institute for Nutrition and Translational Research in Metabolism Maastricht the Netherlands; ^6^ Department of Respiratory Medicine Maastricht University Medical Center Maastricht the Netherlands

**Keywords:** aeroallergens, allergic sensitisation, atopy, cohort studies, hypersensitivity, skin prick test

## Abstract

**Background:**

Allergic sensitisation is traditionally viewed as a stable trait despite studies suggesting that it may vary across life‐course. This study aims to provide longitudinal evidence of allergic sensitisation and its associated factors in the general population.

**Methods:**

Aeroallergen sensitisation was assessed in the Austrian population‐based cohort using repeated skin prick testing (SPT) across 3 visits (mean visit interval, 4.25 ± 0.33 years). Longitudinal sensitisation patterns were characterised as stable nonsensitised, stable sensitised, new‐onset, resolution or fluctuating. We examined demographic, metabolic, behavioural, environmental and immunological factors across age strata (< 18, 18 ≤ 40, 40 ≤ and ≥ 60 years).

**Results:**

Among 5046 individuals with follow‐up and valid SPT in all 3 visits, 54.4% remained stably nonsensitised and 30.6% stably sensitised, 5.6% experienced resolution, 4.8% new onset and 4.6% fluctuating sensitisation. New onset expectedly predominated in < 18 years, fluctuation in 18 ≤ 60 years and resolution in ≥ 60 years, indicating that sensitisation is modifiable and time‐varying. Stable sensitisation was strongly associated with parental allergy and blood eosinophils while cumulative smoking modestly reduced its likelihood. Fluctuation was linked to adiposity (in adults ≥ 40 years old) and environmental exposures. Aeroallergen‐specific analysis showed new‐onset sensitisation was driven by outdoor allergens (ragweed, tree pollens and pets) whereas fluctuation was driven by seasonal pollens.

**Conclusion:**

Although predominantly stable, allergic sensitisation is dynamic and exhibits substantial temporal variation. Resolution and fluctuation occur across all ages and new sensitisation can emerge well into adulthood. These findings challenge the traditional view of fixed sensitisation and suggest it reflects a modifiable phenotype shaped by age and environmental and lifestyle exposures.

## 1 Introduction

Sensitisation, the development of allergen‐specific IgE in response to otherwise benign environmental proteins, underpins allergic disease but often lack symptoms [1]. It is conventionally identified through a positive skin prick test (SPT) or allergen‐specific IgE measurement in serum. This approach fails to capture the dynamic nature of sensitisation which may develop, persist, fluctuate, or resolve over time with life‐course evolution poorly understood.

Longitudinal evidence of sensitisation patterns of aeroallergens is limited. Birth cohorts show new‐onset sensitisation in childhood, while older adults studies suggest declining prevalence with age [2, 3, 4]. Few have examined sensitisation patterns in a single population spanning childhood to late adulthood or assessed how demographic, environmental and immunological factors shape resolution or fluctuation. Likewise, aeroallergen‐specific patterns and polysensitisation remain poorly characterised, despite their clinical relevance.

In this study, we mapped skin prick test (SPT) responses to common inhalant indoor and outdoor aeroallergens, aiming to: (i) quantify the proportion of stable, new‐onset, resolving, and fluctuating sensitisation patterns via age‐stratified, population‐level analyses, (ii) identify total and aeroallergen‐specific patterns and (iii) determine associated factors of these trajectories for a lifespan perspective.

## 2 Methods

### 2.1 Study Design

This study was conducted within the framework of the LEAD (Lung, hEart, sociAl, body) study (clinicalTrials.gov identifier: NCT01727518) which received approval from the local ethics committee of Vienna (Ethikkommission der Stadt Wien; protocol number EK‐11‐117–0711). All procedures in the LEAD study were carried out in accordance with the Declaration of Helsinki, and written informed consent was obtained from all participants and/or legal guardians. The LEAD study is a longitudinal, observational, population‐based cohort study in Austria with follow‐up assessments (2011 to present; mean visit interval, 4.25 ± 0.33 years) [5]. The study reports longitudinal data from visit at recruitment and 2 further follow‐ups and includes individuals with valid SPT (for both histamine and saline controls) at all visits. All participants aged 6‐82 at recruitment were included in the present study.

Our study design involved repeated cross‐sectional assessments of distinct age groups over 3 visits to describe population‐level sensitisation patterns across the lifespan but does not track individual trajectories over decades. Participants were further categorised into five longitudinal sensitisation trajectories based on their patterns of SPT (SPT+, sensitised; and SPT‐, nonsensitised): those with consistently negative sensitisation (stable nonsensitised), consistently positive sensitisation (stable sensitised), those who lost sensitisation over time (resolution), those who developed sensitisation later (new‐onset), and those with nonlinear patterns of change (fluctuating). Fluctuating sensitised individuals were further divided into SPT‐/SPT+/SPT‐ and SPT+/SPT‐/SPT + subgroups for sensitivity analyses.

### 2.2 Measurements

All participants had withdrawn from antihistamines or glucocorticoids (oral and cutaneous) for at least 48 h prior to visits and SPT was performed on the participants' forearms by trained technicians. All reported use of antihistamines in this cohort has half‐lives compatible with a 48‐h washout, making substantial residual pharmacologic effect unlikely. SPT was considered positive in the presence of a reaction ≥ 3 mm diameter for at least any one of the following aeroallergens classified as outdoors (ash tree, tree pollen mix [hazel, birch and alder], grass mix [*Anthoxantum*, *Dactylis*, *Lolium*, *Phleum* and *Poa*], mugwort, ragweed and ribwort); indoors (mites mix [*Dermatophagoides Farinae* and *Dermatophagoides Pternyssinus*] and mildew); and pets (dog and cat dander). All participants underwent SPT with an identical panel of aeroallergens obtained from Alk‐Abello for all visits, ensuring consistency in aeroallergen composition and batch quality. Positive (0.1% histamine) and negative (0.9% sodium chloride) controls were included in the test panels.

Whole‐blood samples were collected from fasted participants (eight‐hours prior to visit) for complete blood cell counts and differential count analysis to obtain measurements for blood eosinophil counts, high‐sensitivity C‐reactive protein (hsCRP), neutrophils and total immunoglobulin E (IgE). IgE measurements were reported only for Visits 2 and 3 as IgE was not measured in participants at Visit 1. Height, weight, and waist circumference were measured with a stadiometer and high precision scale for calculations of body mass index (BMI) by weight/height^2^ and waist‐height ratio. Whole‐body scans were obtained with a Lunar Prodigy (GE Healthcare, USA) Dual x‐ray absorptiometry (DXA) scanner to assess fat mass (FM), and lean mass (LM). Indices of all body composition metrics were additionally calculated as mass/height^2^.

Information on participant's age, smoking status, pack‐years, exposures to dust, passive smoking, socioeconomic status (SES), familial predisposition to allergy, and residence in urban areas or proximal to the main road, was collected by an interview‐based questionnaire. Annual particulate matter (PM_10_) and nitrogen dioxide (NO_2_) concentrations assigned to residential addresses were estimated with the GRAMM/GRAL model (2015–2021) and were reported only for Visits 1 and 2. Socioeconomic status categories were based on a composite score of occupational, educational and income status (SES of participants < 18 years were obtained from their parents or primary guardian) [6]. Further information of any allergy medication (antihistamines and Leukotriene Receptor Antagonist, LTRA) used; respiratory symptoms such as chronic cough, wheeze, nocturnal symptoms (cough and wheeze) and sinonasal disease (nasal polyps and sinusitis); physician's diagnosis of eczema, rhino‐conjunctivitis and allergy; and history of asthma were self‐reported.

### 2.3 Statistical Analysis

All statistical analyses were performed in R (version 4.3.0; R foundation for Statistical Computing, Vienna, Austria), with the level of significance set at < 5%. Data are presented as mean ± standard deviation or relative frequency (percentage of group). Analysis of variance (ANOVA) and chi‐squared tests were used for comparisons of nominal and binary variables respectively in between‐group comparisons. T‐tests and Fisher exact tests with false‐discovery rate corrections were applied in post‐hoc analysis. Total and aeroallergen‐specific sensitisation patterns across time were visualised with alluvial plots and heatmaps. To assess for potential effect modification by age, all primary analyses were repeated with stratification by distinct baseline age groups (< 18, 18 ≤ 40, 40 ≤ 60 and ≥ 60 years). Stepwise multinomial multivariable regression models (forced entry and backwards) were used to determine risk factors associated with sensitisation patterns. Among variables, BMI was removed due to collinearity or large missing values (> 10%).

Control charts (p‐charts) were used to visualise the proportion of valid histamine and saline controls per monthly batch (*x*‐axis). The horizontal central line (CL) observed the overall average validity rate across all batches. The upper and lower control limits (UCL and LCL respectively) define the range of variation expected by change based on binomial distribution assumptions. Points highlighted in red points indicate batches flagged as rule violations (i.e., beyond the control limits or part of a nonrandom sequence), suggesting deviations from expected random variations.

## 3 Results

### 3.1 Sensitisation at Each Visit and Test Reliability

SPT controls were consistently valid across phases (> 98%). Although 8 batches exceeded statistical limits and 40 runs violated run rules, these deviations were rare and isolated (Supporting Information S1: Figure S1). All histamine and saline controls were within acceptable thresholds, supporting minimal batch‐to‐batch variability and ensured that the observed changes in sensitisation patterns reflected biological rather than technical variation.

Among 5046 eligible participants (48.3% males; mean age 46.2 ± 17.6 years; and 19.1% current smoking), sensitised individuals were more often males had lower fat mass index (FMI) and higher lean mass index (LMI), fewer current smokers and lower pack years, and reported higher parental allergies (Table 1). Eosinophils and IgE were significantly higher in the sensitised groups at Visits 2 and 3 (*p* < 0.001). Lifestyle and environmental differences were minimal. In sensitised individuals, body composition and smoking exposures increased with age (Table 1).

**TABLE 1:** Characteristics of sensitised individuals in different age strata.

		Visit 1				Visit 2				Visit 3			
		< 18	18 ≤ 40	40 ≤ 60	≥ 60	< 18	18 ≤ 40	40 ≤ 60	≥ 60	< 18	18 ≤ 40	40 ≤ 60	≥ 60
Total *N*, %	SPT‐	289 (64.1)	637 (54.0)	1329 (61.6)	924 (73.4)	253 (56.1)	602 (51.1)	1268 (58.8)	902 (71.7)	256 (56.8)	638 (54.1)	1354 (62.7)	969 (77.0)
	SPT+	162 (35.9)	542 (46.0)	829 (38.4)	334 (26.6)	198 (43.9)	577 (48.9)	890 (41.2)	356 (28.3)	195 (43.2)	541 (45.9)	804 (37.3)	289 (23.0)
Males, %	SPT‐	127 (43.9)	273 (42.9)	595 (44.8)	441 (47.7)	111 (43.9)	254 (42.2)	570 (45.0)	428 (47.5)	111 (43.4)	277 (43.4)	611 (45.1)	462 (47.7)
	SPT+	89 (54.9)	337 (62.2)	449 (54.2)	178 (53.3)	105 (53.0)	356 (61.7)	474 (53.3)	191 (53.7)	105 (53.8)	333 (61.6)	433 (53.9)	157 (54.3)
	*p*	0.032	< 0.001	< 0.001	0.093	0.066	< 0.001	< 0.001	0.055	0.035	< 0.001	< 0.001	0.055
Age, years	SPT‐	12.1 ± 3.3	30.6 ± 6.1	50.6 ± 5.5	67.7 ± 4.9	16.6 ± 3.4	34.8 ± 6.1	54.8 ± 5.5	71.8 ± 5.0	20.9 ± 3.5	39.1 ± 6.2	59.1 ± 5.4	76.2 ± 4.9
	SPT+	12.6 ± 3.3	30.1 ± 6.3	49.6 ± 5.3	66.8 ± 4.8	16.4 ± 3.3	34.5 ± 6.2	53.8 ± 5.4	71.1 ± 4.6	20.8 ± 3.4	38.7 ± 6.3	57.9 ± 5.3	75.0 ± 4.7
	*p*	0.141	0.236	< 0.001	0.007	0.547	0.535	< 0.001	0.014	0.736	0.318	< 0.001	< 0.001
Waist‐height, ratio	SPT‐	0.5 ± 0.1	0.5 ± 0.1	0.6 ± 0.1	0.6 ± 0.1	0.5 ± 0.1	0.5 ± 0.1	0.6 ± 0.1	0.6 ± 0.1	0.5 ± 0.1	0.5 ± 0.1	0.6 ± 0.1	0.6 ± 0.1
	SPT+	0.5 ± 0.1	0.5 ± 0.1	0.5 ± 0.1	0.6 ± 0.1	0.5 ± 0.1	0.5 ± 0.1	0.6 ± 0.1	0.6 ± 0.1	0.5 ± 0.1	0.5 ± 0.1	0.6 ± 0.1	0.6 ± 0.1
	*p*	0.733	0.480	0.011	0.461	0.806	0.066	0.084	0.306	0.882	0.294	0.001	0.478
BMI, kg/m^2^	SPT‐	18.6 ± 3.3	23.9 ± 4.3	26.3 ± 4.7	27.4 ± 4.2	21.1 ± 4.0	24.5 ± 4.5	26.6 ± 4.8	27.4 ± 4.3	23.4 ± 4.5	25.2 ± 4.6	27.0 ± 4.9	27.2 ± 4.3
	SPT+	19.2 ± 3.4	23.7 ± 3.9	25.9 ± 4.3	27.1 ± 4.1	21.7 ± 3.7	24.4 ± 4.1	26.5 ± 4.6	27.3 ± 4.3	23.3 ± 4.2	25.0 ± 4.3	26.4 ± 4.6	27.1 ± 4.4
	*p*	0.124	0.591	0.036	0.305	0.134	0.719	0.356	0.921	0.892	0.501	0.004	0.853
FMI, kg/m^2^	SPT‐	5.4 ± 2.2	7.2 ± 3.0	9.1 ± 3.5	10.1 ± 3.3	6.4 ± 2.8	7.8 ± 3.2	9.5 ± 3.6	10.3 ± 3.4	7.1 ± 3.2	8.2 ± 3.4	9.9 ± 3.7	10.2 ± 3.4
	SPT+	5.6 ± 2.4	6.8 ± 2.8	8.4 ± 3.0	9.8 ± 3.2	6.4 ± 2.7	7.3 ± 2.9	9.0 ± 3.3	10.0 ± 3.2	7.0 ± 3.0	7.7 ± 3.1	9.1 ± 3.4	10.0 ± 3.2
	*p*	0.438	0.006	< 0.001	0.132	0.866	0.005	0.003	0.293	0.531	0.013	< 0.001	0.279
LMI, kg/m^2^	SPT‐	12.7 ± 1.9	15.7 ± 2.3	16.3 ± 2.3	16.5 ± 2.1	14.0 ± 2.0	15.9 ± 2.3	16.3 ± 2.3	16.3 ± 2.0	15.2 ± 2.2	16.0 ± 2.3	16.3 ± 2.3	16.1 ± 2.0
	SPT+	13.1 ± 2.2	16.1 ± 2.3	16.5 ± 2.3	16.5 ± 2.1	14.6 ± 2.3	16.2 ± 2.2	16.5 ± 2.3	16.4 ± 2.2	15.5 ± 2.3	16.4 ± 2.3	16.3 ± 2.3	16.3 ± 2.1
	*p*	0.089	0.004	0.071	0.978	0.004	0.004	0.052	0.226	0.301	0.013	0.360	0.143
hsCRP, mg/dL	SPT‐	0.8 ± 2.7	1.6 ± 3.1	1.9 ± 2.8	2.3 ± 2.8	3.7 ± 4.3	3.5 ± 3.0	3.7 ± 4.0	3.8 ± 3.6	3.4 ± 3.1	3.0 ± 2.1	3.7 ± 3.4	3.7 ± 3.2
	SPT+	1.1 ± 2.7	1.5 ± 3.7	1.9 ± 3.1	2.4 ± 4.7	3.1 ± 1.1	3.5 ± 2.9	3.5 ± 2.1	3.9 ± 4.5	2.8 ± 2.2	3.0 ± 3.5	3.4 ± 2.7	3.6 ± 2.5
	*p*	0.393	0.662	0.704	0.657	0.038	0.844	0.044	0.604	0.015	0.839	0.092	0.578
Neutrophils, cells/L	SPT‐	2.8 ± 0.6	3.9 ± 1.5	4.0 ± 1.4	3.9 ± 1.2	3.7 ± 1.5	3.8 ± 1.4	3.9 ± 1.4	4.0 ± 1.3	3.8 ± 1.5	3.7 ± 1.4	3.8 ± 1.5	4.1 ± 1.3
	SPT+	2.7 ± 0.6	3.8 ± 1.5	3.8 ± 1.4	3.9 ± 1.2	3.9 ± 1.4	3.7 ± 1.4	3.8 ± 1.3	3.9 ± 1.3	3.6 ± 1.3	3.6 ± 1.5	3.7 ± 1.4	3.9 ± 1.2
	*p*	0.538	0.128	0.006	0.502	0.285	0.813	0.162	0.039	0.104	0.268	0.033	0.006
Eosinophils, cells/μL	SPT‐	3.7 ± 1.4	137.8 ± 122.3	147.4 ± 108.7	147.5 ± 112.1	163.7 ± 164.5	155.2 ± 143.2	165.2 ± 119.0	170.3 ± 134.0	126.2 ± 112.2	148.3 ± 120.5	162.8 ± 174.7	163.5 ± 138.1
	SPT+	3.6 ± 1.5	175.1 ± 146.6	171.7 ± 141.8	192.2 ± 179.8	244.1 ± 217.6	183.3 ± 130.3	186.6 ± 130.5	201.1 ± 184.8	203.7 ± 150.7	171.2 ± 127.8	166.7 ± 118.7	206.9 ± 159.2
	*p*	0.726	< 0.001	< 0.001	< 0.001	< 0.001	< 0.001	< 0.001	0.005	< 0.001	0.002	0.535	< 0.001
IgE, kU/L	SPT‐	—	—	—	—	70.9 ± 187.6	41.6 ± 118.9	49.6 ± 126.1	70.8 ± 287.1	45.4 ± 71.8	41.9 ± 144.4	61.4 ± 277.7	85.0 ± 365.8
	SPT+	—	—	—	—	285.5 ± 648.3	141.5 ± 220.0	150.5 ± 391.6	134.7 ± 277.5	332.4 ± 1371.9	138.0 ± 252.6	137.1 ± 305.4	150.8 ± 391.6
	*p*	—	—	—	—	< 0.001	< 0.001	< 0.001	0.001	0.004	< 0.001	< 0.001	0.012
Use of any allergy medication, %	SPT‐	3 (1.0)	1 (0.2)	20 (1.5)	19 (2.1)	1 (0.4)	7 (1.2)	18 (1.4)	19 (2.1)	1 (0.4)	12 (1.9)	30 (2.2)	20 (2.1)
	SPT+	14 (8.6)	47 (8.7)	48 (5.8)	13 (3.9)	15 (7.6)	47 (8.1)	45 (5.1)	20 (5.6)	28 (14.4)	108 (20.0)	86 (10.7)	28 (9.7)
	*p*	< 0.001	< 0.001	< 0.001	0.104	< 0.001	< 0.001	< 0.001	0.002	< 0.001	< 0.001	< 0.001	< 0.001
Sinonasal disease, %	SPT‐	10 (3.5)	14 (2.2)	57 (4.3)	36 (3.9)	21 (8.3)	63 (10.5)	154 (12.1)	140 (15.5)	15 (5.9)	86 (13.5)	194 (14.3)	133 (13.7)
	SPT+	3 (1.9)	12 (2.2)	39 (4.7)	16 (4.8)	8 (4.0)	77 (13.3)	152 (17.1)	61 (17.1)	10 (5.1)	77 (14.2)	120 (14.9)	120 (14.9)
	*p*	0.493	1.000	0.728	0.587	0.102	0.150	0.002	0.536	0.898	0.773	0.751	0.751
Allergy, %	SPT‐	13 (4.5)	71 (11.2)	208 (15.7)	140 (15.2)	25 (9.9)	82 (13.6)	208 (16.4)	119 (13.3)	33 (12.9)	108 (16.9)	268 (19.8)	145 (15.0)
	SPT+	65 (40.1)	327 (60.3)	528 (63.8)	163 (49.1)	110 (55.6)	406 (70.6)	588 (66.2)	182 (51.7)	123 (63.1)	371 (68.6)	539 (67.0)	164 (56.7)
	*p*	< 0.001	< 0.001	< 0.001	< 0.001	< 0.001	< 0.001	< 0.001	< 0.001	< 0.001	< 0.001	< 0.001	< 0.001
Rhino‐conjunctivitis, %	SPT‐	6 (2.1)	38 (6.0)	86 (6.5)	50 (5.4)	10 (4.0)	22 (3.7)	54 (4.3)	29 (3.2)	18 (7.0)	51 (8.0)	88 (6.5)	34 (3.5)
	SPT+	66 (40.7)	327 (60.3)	502 (60.6)	149 (44.6)	104 (52.5)	370 (64.1)	525 (59.0)	161 (45.2)	129 (66.2)	369 (68.2)	490 (60.9)	141 (48.8)
	*p*	< 0.001	< 0.001	< 0.001	< 0.001	< 0.001	< 0.001	< 0.001	< 0.001	< 0.001	< 0.001	< 0.001	< 0.001
Eczema, %	SPT‐	21 (7.3)	62 (9.7)	194 (14.6)	116 (12.6)	18 (7.1)	49 (8.2)	153 (12.1)	85 (9.6)	18 (7.2)	65 (10.3)	181 (13.5)	85 (8.9)
	SPT+	24 (14.8)	116 (21.4)	204 (24.6)	63 18.9)	33 (18.1)	113 (21.8)	194 (24.4)	56 (16.6)	41 (23.7)	120 (25.4)	197 (27.7)	51 (20.2)
	*p*	0.005	< 0.001	< 0.001	0.001	0.001	< 0.001	< 0.001	0.001	< 0.001	< 0.001	< 0.001	< 0.001
Nocturnal symptoms, %	SPT‐	8 (2.8)	21 (3.3)	49 (3.7)	36 (3.9)	2 (0.8)	6 (1.0)	13 (1.0)	13 (1.4)	6 (2.3)	11 (1.7)	43 (3.2)	37 (3.8)
	SPT+	9 (5.6)	23 (4.2)	43 (5.2)	18 (5.4)	3 (1.5)	12 (2.1)	19 (2.1)	7 (2.0)	5 (2.6)	17 (3.1)	29 (3.6)	12 (4.2)
	*p*	0.217	0.484	0.117	0.319	0.782	0.201	0.055	0.674	1.000	0.161	0.678	0.933
Chronic cough, %	SPT‐	7 (2.4)	30 (4.7)	98 (7.4)	79 (8.6)	3 (1.2)	10 (1.7)	64 (5.0)	64 (7.1)	5 (8.2)	15 (13.2)	42 (11.0)	39 (11.4)
	SPT+	2 (1.2)	30 (5.5)	54 (6.6)	26 (7.8)	1 (0.5)	29 (5.0)	53 (6.0)	27 (7.6)	3 (9.1)	15 (12.6)	30 (14.0)	15 (14.7)
	*p*	0.609	0.619	0.516	0.753	0.793	0.002	0.412	0.860	1.000	1.000	0.344	0.476
Wheeze, %	SPT‐	10 (3.5)	45 (7.1)	100 (7.5)	74 (8.0)	5 (2.0)	15 (2.5)	56 (4.4)	29 (3.2)	10 (3.9)	10 (1.6)	70 (5.2)	40 (4.1)
	SPT+	14 (8.6)	55 (10.1)	80 (9.7)	31 (9.3)	3 (1.5)	30 (5.2)	53 (6.0)	20 (5.6)	9 (4.6)	30 (5.5)	53 (6.6)	12 (4.2)
	*p*	0.033	0.074	0.098	0.545	0.993	0.023	0.132	0.068	0.893	< 0.001	0.200	1.000
Asthma, %	SPT‐	4 (1.6)	18 (2.7)	47 (3.2)	29 (2.5)	4 (1.6)	16 (2.7)	41 (3.3)	23 (2.6)	8 (3.1)	21 (0.3)	49 (3.6)	37 (3.8)
	SPT+	14 (11.1)	75 (13.7)	115 (12.7)	30 (8.7)	22 (11.3)	79 (14.4)	113 (13.4)	31 (9.1)	25 (12.8)	86 (15.9)	98 (12.2)	28 (9.7)
	*p*	< 0.001	< 0.001	< 0.001	< 0.001	< 0.001	< 0.001	< 0.001	< 0.001	< 0.001	< 0.001	< 0.001	< 0.001
Never smokers, %	SPT‐	279 (97.2)	326 (51.2)	498 (37.5)	408 (44.2)	222 (88.1)	285 (49.9)	454 (37.9)	386 (45.2)	191 (76.7)	268 (46.8)	430 (35.1)	366 (42.0)
	SPT+	157 (96.9)	287 (53.0)	375 (45.2)	159 (47.6)	183 (92.4)	288 (52.7)	389 (46.0)	161 (48.5)	158 (81.4)	254 (52.2)	334 (45.5)	122 (46.9)
	*p*	0.87	0.58	< 0.001	0.31	0.17	0.82	< 0.001	0.34	0.27	0.092	< 0.001	0.18
Former smokers, %	SPT‐	—	143 (22.4)	487 (36.6)	389 (42.1)	6 (2.4)	153 (26.8)	467 (39.0)	377 (44.1)	18 (7.2)	183 (31.9)	540 (44.1)	419 (48.1)
	SPT+	—	114 (21.0)	283 (34.1)	149 (44.6)	2 (1.0)	135 (24.7)	316 (37.4)	145 (43.7)	12 (6.2)	139 (28.5)	284 (38.7)	123 (47.3)
	*p*	—	0.61	0.26	0.47	0.46	0.47	0.48	0.94	0.81	0.26	0.022	0.88
Current smokers, %	SPT‐	8 (2.8)	168 (26.4)	344 (25.9)	127 (13.7)	24 (9.5)	133 (23.3)	277 (23.1)	91 (10.7)	40 (16.1)	122 (21.3)	255 (20.8)	86 (9.9)
	SPT+	5 (3.1)	141 (26.0)	171 (20.6)	26 (7.8)	13 (6.6)	123 (22.5)	141 (16.7)	26 (7.8)	24 (12.4)	94 (19.3)	116 (15.8)	15 (5.8)
	*p*	1	0.94	0.006	0.006	0.34	0.82	< 0.001	0.18	0.34	0.47	0.007	0.056
Pack‐years	SPT‐	0.0 ± 0.2	3.7 ± 6.8	12.4 ± 17.7	13.9 ± 22.1	0.2 ± 0.9	4.0 ± 7.6	12.4 ± 18.0	13.8 ± 23.6	0.5 ± 2.0	4.7 ± 8.5	14.2 ± 19.9	14.3 ± 24.0
	SPT+	0.0 ± 0.0	2.7 ± 5.1	9.4 ± 15.5	10.4 ± 17.7	0.1 ± 0.4	3.3 ± 6.1	9.8 ± 15.6	11.2 ± 18.6	0.3 ± 1.2	3.8 ± 7.8	10.0 ± 17.2	12.6 ± 24.2
	*p*	0.136	0.004	< 0.001	0.004	0.096	0.106	0.001	0.050	0.189	0.075	< 0.001	0.322
Passive smoking, %	SPT‐	166 (57.4)	434 (68.1)	879 (66.1)	590 (63.9)	156 (61.7)	435 (72.3)	896 (70.7)	608 (67.4)	165 (64.5)	485 (76.0)	992 (73.3)	677 (69.9)
	SPT+	88 (54.3)	356 (65.7)	531 (64.1)	209 (62.6)	116 (58.6)	408 (70.7)	609 (68.4)	242 (68.0)	123 (63.1)	387 (71.5)	567 (70.5)	206 (71.3)
	*p*	0.588	0.407	0.345	0.727	0.572	0.600	0.287	0.898	0.840	0.093	0.185	0.698
Urbanicity, %	SPT‐	255 (88.2)	563 (88.4)	1086 (81.7)	710 (76.8)	217 (86.5)	493 (83.1)	1007 (80.0)	669 (75.3)	221 (88.0)	476 (79.2)	1015 (77.2)	717 (76.4)
	SPT+	134 (82.7)	487 (89.9)	659 (79.5)	263 (78.7)	163 (82.3)	497 (87.0)	687 (77.7)	268 (76.1)	157 (81.8)	445 (86.1)	610 (77.7)	214 (76.2)
	*p*	0.136	0.477	0.222	0.525	0.283	0.074	0.210	0.825	0.086	0.003	0.849	1.000
Residence near main road, %	SPT‐	162 (56.8)	393 (62.4)	767 (58.4)	507 (55.7)	146 (57.9)	383 (63.6)	659 (52.0)	429 (47.9)	151 (59.4)	323 (50.9)	563 (41.7)	412 (43.0)
	SPT+	89 (55.6)	370 (68.4)	460 (55.7)	175 (52.9)	95 (48.0)	365 (63.3)	447 (50.2)	166 (46.9)	109 (56.5)	267 (49.4)	301 (37.4)	109 (38.0)
	*p*	0.882	0.037	0.232	0.409	0.045	0.945	0.450	0.788	0.593	0.646	0.055	0.148
Exposure to dust, %	SPT‐	5 (3.7)	111 (17.5)	301 (22.7)	201 (21.8)	16 (6.9)	163 (27.1)	376 (29.7)	280 (31.2)	37 (14.5)	204 (32.1)	487 (36.0)	345 (36.1)
	SPT+	0 (0.0)	89 (16.4)	181 (21.9)	77 (23.1)	8 (4.5)	152 (26.3)	278 (31.2)	108 (30.4)	28 (14.5)	164 (30.3)	283 (35.2)	101 (35.3)
	*p*	0.165	0.695	0.723	0.679	0.425	0.827	0.459	0.846	1.000	0.558	0.751	0.866
Socioeconomic status, score	SPT‐	5.9 ± 2.9	12.4 ± 3.8	12.8 ± 3.6	12.2 ± 3.0	9.2 (2.9)	14.5 (3.1)	14.2 (2.8)	13.3 (2.4)	11.4 (3.1)	15.6 (2.6)	14.5 (2.6)	13.7 (2.2)
	SPT+	5.6 ± 2.8	12.2 ± 4.1	13.8 ± 3.4	12.4 ± 3.1	9.1 (3.0)	14.7 (2.9)	14.7 (2.8)	13.4 (2.4)	11.5 (2.9)	15.5 (2.7)	15.1 (2.5)	13.9 (2.2)
	*p*	0.438	0.272	< 0.001	0.450	0.941	0.203	< 0.001	0.492	0.856	0.421	< 0.001	0.188
Socioeconomic status, low, %	SPT‐	205 (70.9)	108 (17.0)	183 (13.8)	108 (11.7)	71 (28.1)	26 (4.3)	32 (2.5)	22 (2.5)	32 (12.5)	11 (1.7)	27 (2.0)	10 (1.0)
	SPT+	118 (72.8)	122 (22.5)	70 (8.4)	41 (12.3)	58 (29.3)	16 (2.8)	24 (2.7)	8 (2.3)	21 (10.8)	15 (2.8)	13 (1.6)	2 (0.7)
	*p*	0.748	0.020	< 0.001	0.853	0.856	0.202	0.911	1.000	0.679	0.314	0.632	0.858
PM_10_, μg/m^3^	SPT‐	20.8 ± 1.4	21.1 ± 1.2	20.9 ± 1.2	20.7 ± 1.1	20.7 ± 1.3	21.0 ± 1.2	20.7 ± 1.2	20.6 ± 1.1	—	—	—	—
	SPT+	20.8 ± 1.2	21.2 ± 1.3	20.9 ± 1.2	20.6 ± 1.0	20.8 ± 1.2	21.1 ± 1.3	20.8 ± 1.2	20.6 ± 1.1	—	—	—	—
	*p*	0.893	0.342	0.449	0.116	0.948	0.386	0.139	0.712	—	—	—	—
NO_2_, μg/m^3^	SPT‐	20.3 ± 3.9	20.8 ± 4.2	20.8 ± 4.0	21.1 ± 4.3	19.5 ± 3.1	20.1 ± 3.4	20.0 ± 3.5	20.4 ± 3.9	—	—	—	—
	SPT+	21.0 ± 3.9	20.8 ± 4.2	21.2 ± 4.4	21.5 ± 4.3	19.9 ± 3.8	20.1 ± 3.6	20.2 ± 3.7	20.4 ± 3.6	—	—	—	—
	*p*	0.130	0878	0.077	0.270	0.201	0.993	0.163	0.736	—	—	—	—
Parental allergies, %	SPT‐	138 (48.6)	149 (24.3)	141 (11.2)	37 (4.4)	—	—	—	—	—	—	—	—
	SPT+	109 (69.0)	213 (41.4)	139 (18.1)	20 (6.7)	—	—	—	—	—	—	—	—
	*p*	< 0.001	< 0.001	< 0.001	0.147	—	—	—	—	—	—	—	—

*Note:* Data is presented as mean ± standard deviations or frequency (%). Age strata is based on age at baseline. Significance is considered where *p* < 0.05.

Abbreviations: BMI, body mass index; FMI, fat mass index; hsCRP, high‐sensitivity C‐reactive protein; IgE, immunoglobulin E; LMI, lean mass index; NO_2_, nitrogen dioxide.

**FIGURE 1:**
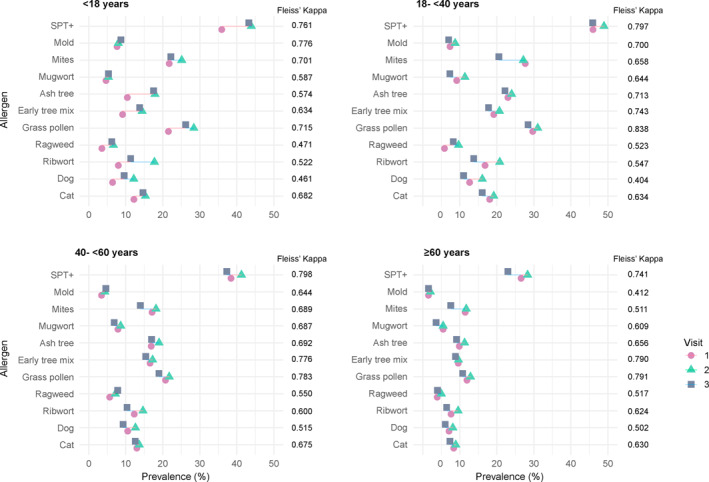
Allergen positivity over time by age strata. SPT+, positive skin prick test.

**FIGURE 2:**
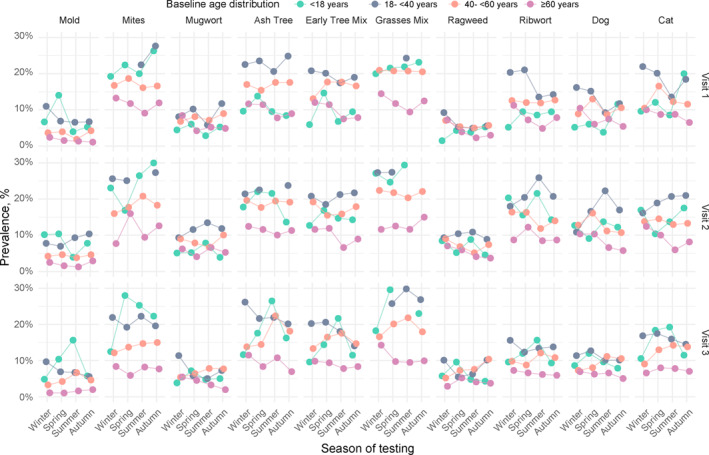
Seasonal allergen‐specific positivity stratified by age groups in each visit. Winter, December–February; Spring, March–May; Summer, June–August; and Autumn, September–November.

SPT positivity fluctuated across visits with moderate reproducibility (Fleiss' κ 0.741–0.798; Figure 1) and minimal net change (−0.01%; Cohen's H, −0.02, McNemar's *p* = 0.106). Outdoor allergens were generally stable although ragweed (Fleiss' κ 0.471–0.523) and ribwort (Fleiss' κ 0.522–0.624) were more variable. Grass pollen was most consistent (Fleiss' κ 0.715–0.838) and most prevalent (> 50% across ages) with strong seasonal peaks (spring/summer) (Figure 2). Mite sensitisation declined, whereas mold remained low and stable. Pet sensitisation increased in < 18 years but decreased in 18 ≤ 40 years, with cat sensitisation consistently higher than dog (Figure 1).

### 3.2 Longitudinal Sensitisation Patterns and Associated Factors

**FIGURE 3:**
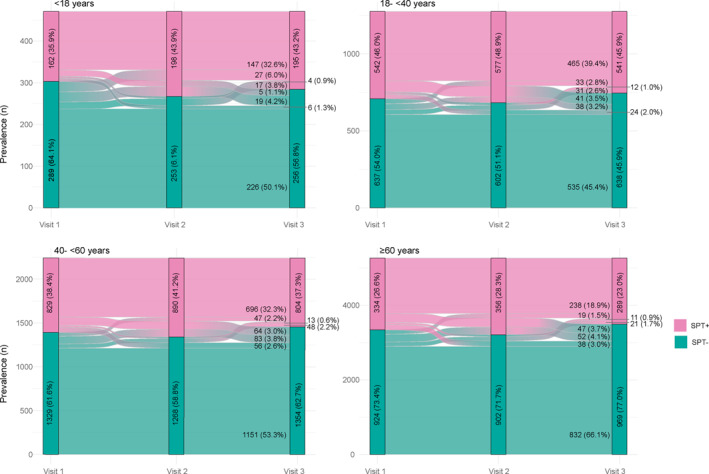
Alluvial plot of longitudinal SPT patterns over time in the general population. SPT+, positive skin prick test; and SPT−, negative skin prick test.

The distribution of longitudinal sensitisation patterns within each age strata is visualised in Figure 3 and Supporting Information S1: Tables S1–S4. Stable SPT−increased with age (65.9% in ≥ 60 vs. 50.3% in < 18 years), while stable SPT + peaked in 18 ≤ 40 years (34.7%). New‐onset was most common in < 18 years (8.9%), while resolution was constant across ages (7.0%–8.2%). Fluctuating sensitisation ranged 4%–8% across strata, peaking in 40 ≤ 60 years (8.3%). Sensitive analysis using latent class analysis (LCA), performed using SPT positivity at 3 visits as indicators, supported a 2‐class solution corresponding to stable positive and stable negative sensitisation in all age‐stratified groups. Models with more classes up to 5 fit worse (Bayesian Information Criterion 12522.1 vs. 12624.5) and aligned poorly with predefined patterns (weighted κ −0.2 to −0.143), indicating resolution, new‐onset and fluctuation were infrequent relative to persistence (Supporting Information S1: Table S5).

In < 18 years, stable SPT + occurs more often in males (55.0%), with higher eosinophils, asthma (8.6%) and parental allergies (69.9%). In 40 ≤ 60 years, stable SPT + remained eosinophilic and asthma‐associated; resolution, new‐onset and fluctuations groups were more prominent among women and associated with higher smoking and FMI. In ≥ 60 years, resolution and fluctuating were linked with smoking and higher adiposity whereas persistent sensitisation remained eosinophilic. Stable SPT‐individuals showed higher smoking and BMI from early adulthood. Body composition also differed for all age strata: stable SPT + had the lowest BMI and FMI, whereas the fluctuating group had the highest BMI and FMI although no clear subgroup explanation (SPT+/SPT‐/SPT + vs. SPT‐/SPT+/SPT‐) accounting for these body composition differences (Supporting Information S1: Table S6). Within the fluctuation group, low overall positivity with batch‐to‐batch variability was observed with excellent control performance (Supporting Information S1: Figure S2). Allergen‐specific sub‐analyses of the fluctuation group (Supporting Information S1: Figure S3) indicated that fluctuation disproportionately involved seasonal allergens such as ragweed, ribwort, and tree pollens, whereas perennial allergens such as grass and mites were largely stable. Allergy medication use was generally highest in the stable SPT+ (∼10% of participants < 40 years) and fluctuating groups, with the exception observed in those 40 ≤ 60 years where stable SPT+ and new‐onset predominated. Sensitised groups also reported more allergy, rhino‐conjunctivitis and eczema whereas wheeze and chronic cough were less discriminative between SPT patterns.

Multinomial stepwise regression (Table 2) showed strong associations of parental allergies and eosinophils with stable SPT + across ages, notably in 18 ≤ 40 years (corresponding OR [95% CI]: 3.00 [2.25, 4.00] and 1.00 [1.00, 1.00], *p* < 0.001) and in 40 ≤ 60 years, although in this group smoking exposure reduced the likelihood of stable SPT+ (pack‐years, OR 0.99 [95% CI 0.98, 0.99], *p* < 0.001). In ≥ 60 years, cumulative smoking similarly attenuated stable SPT+ (OR 0.99 [95% CI 0.98, 1.00], *p* = 0.050). Fluctuating sensitisation showed age‐specific associations: residence near a main road (OR 2.27 [95% CI 1.13, 4.54], *p* = 0.021) in 18 ≤ 40 years; waist–to‐height ratio and PM_10_ (corresponding OR [95% CI]: 66.5 [7.79, 566.78] and 1.21 [1.08, 1.36], *p* ≤ 0.001) in 40 ≤ 60 years; and NO_2_ (OR 1.06 [95% CI 1.01, 1.12], *p* = 0.029) in ≥ 60 years. Resolution showed weaker associations overall except hsCRP in ≥ 60 years (OR 1.68 [95% CI 1.02, 1.12], *p* = 0.009). In sensitivity analyses, smoking status showed small inverse associations with stable SPT+ (Supporting Information S1: Table S7). No temporal interactions were observed.

**TABLE 2:** Multinomial stepwise regression of risk factors against sensitisation patterns in different age strata.

	Stable sensitisation		Resolution		New‐onset		Fluctuation	
	OR (95% CI)	*p*‐value	OR (95% CI)	*p*‐value	OR (95% CI)	*p*‐value	OR (95% CI)	*p*‐value
< 18								
LMI, kg/m^2^	1.133 (1.016, 1.263)	0.024	1.102 (0.825, 1.472)	0.512	0.902 (0.751, 1.083)	0.267	1.198 (0.988, 1.453)	0.067
Packyears	0.022 (0, 3.418)	0.138	1.23 (0.125, 12.116)	0.859	0 (0, 0)	0.000	0 (0, 0)	0.000
Eosinophils, cells/μL	1.003 (1.002, 1.005)	0.000	0.997 (0.991, 1.004)	0.389	1.001 (0.999, 1.003)	0.357	0.999 (0.996, 1.003)	0.661
Parental allergy (ref. no)	2.842 (1.795, 4.5)	0.000	1.402 (0.414, 4.749)	0.587	1.597 (0.826, 3.089)	0.164	1.111 (0.467, 2.643)	0.813
18–40								
Packyears	0.959 (0.937, 0.981)	0.000	0.981 (0.94, 1.025)	0.398	0.986 (0.943, 1.031)	0.531	1.009 (0.969, 1.05)	0.679
Eosinophils, cells/μL	1.002 (1.001, 1.003)	0.000	1 (0.998, 1.003)	0.763	0.998 (0.995, 1.001)	0.180	0.998 (0.995, 1.002)	0.323
Females (ref. males)	0.354 (0.27, 0.466)	0.000	0.67 (0.396, 1.133)	0.135	0.811 (0.477, 1.379)	0.439	0.675 (0.372, 1.224)	0.195
Residence proximal to road (ref. no)	1.421 (1.076, 1.876)	0.013	1.438 (0.824, 2.509)	0.201	0.932 (0.548, 1.585)	0.796	2.265 (1.132, 4.535)	0.021
Parental allergy (ref. no)	2.995 (2.245, 3.996)	0.000	1.246 (0.687, 2.258)	0.469	1.202 (0.662, 2.181)	0.546	1.273 (0.65, 2.491)	0.482
40–60								
Waist‐to‐height, ratio	0.148 (0.036, 0.609)	0.008	6.372 (0.803, 50.579)	0.080	0.227 (0.02, 2.564)	0.231	66.453 (7.791, 566.773)	0.000
Packyears	0.986 (0.979, 0.993)	0.000	0.993 (0.982, 1.005)	0.252	0.988 (0.974, 1.003)	0.109	0.999 (0.988, 1.01)	0.841
Eosinophils, cells/μL	1.002 (1.001, 1.003)	0.000	1.001 (1, 1.003)	0.058	1.001 (0.999, 1.003)	0.269	1.001 (0.999, 1.003)	0.394
SES, score	1.085 (1.054, 1.117)	0.000	0.989 (0.94, 1.042)	0.682	0.968 (0.915, 1.025)	0.263	1.011 (0.954, 1.071)	0.721
Females (ref. males)	0.61 (0.498, 0.747)	0.000	0.755 (0.511, 1.115)	0.158	0.806 (0.522, 1.243)	0.329	0.878 (0.569, 1.355)	0.557
Urbanicity (ref. rural)	0.842 (0.656, 1.081)	0.178	0.612 (0.395, 0.948)	0.028	1.001 (0.579, 1.73)	0.997	0.56 (0.347, 0.904)	0.018
PM_10_, μg/m^3^	1.067 (0.988, 1.153)	0.099	1.025 (0.919, 1.142)	0.659	0.925 (0.817, 1.047)	0.217	1.21 (1.078, 1.357)	0.001
Parental allergy (ref. no)	1.95 (1.479, 2.571)	0.000	1.478 (0.85, 2.567)	0.166	1.211 (0.639, 2.295)	0.558	1.689 (0.935, 3.05)	0.082
≥ 60								
Waist‐to‐height, ratio	0.252 (0.025, 2.494)	0.239	0.773 (0.028, 21.512)	0.880	1.141 (0.398, 3.27)	0.807	246.115 (7.618, 7950.859)	0.002
Packyears	0.99 (0.98, 1)	0.050	0.985 (0.969, 1.001)	0.066	1.004 (0.99, 1.019)	0.558	1.005 (0.993, 1.018)	0.373
hsCRP, mg/dL	0.972 (0.912, 1.035)	0.369	1.068 (1.017, 1.121)	0.009	1.039 (0.959, 1.127)	0.348	1.029 (0.957, 1.106)	0.447
Eosinophils, cells/μL	1.003 (1.002, 1.004)	0.000	1.002 (1, 1.003)	0.058	0.998 (0.994, 1.002)	0.252	1.001 (0.998, 1.003)	0.612
Former smoking (ref. never)	1.125 (0.778, 1.627)	0.531	1.709 (0.974, 2.998)	0.062	1.732 (0.788, 3.805)	0.171	1.164 (0.599, 2.264)	0.654
Current smoking (ref. never)	0.522 (0.281, 0.969)	0.039	0.994 (0.41, 2.41)	0.989	0.425 (0.09, 2.013)	0.281	1.213 (0.512, 2.872)	0.661
NO_2_, μg/m^3^	1 (0.966, 1.035)	0.996	1.073 (1.025, 1.124)	0.002	1.003 (0.931, 1.08)	0.942	1.061 (1.006, 1.119)	0.029

*Note:* Estimates are odds ratios (OR) with 95% confidence intervals (CI) obtained from entry and backwards stepwise regression models. Sensitisation patterns were referenced to stable nonsensitised individuals. Significance is considered where *p* < 0.05.

Abbreviations: BMI, body mass index; FMI, fat mass index; hsCRP, high‐sensitivity C‐reactive protein; IgE, immunoglobulin E; LMI, lean mass index; NO_2_, nitrogen dioxide; SES, socioeconomic status.

### 3.3 Allergen‐Specific Patterns and Polysensitisation

Among those sensitised at baseline, 59.2% developed ≥ 1 additional sensitisation during follow‐up. The probability of acquiring new sensitisations differed significantly according to the type of primary sensitisation (*p* < 0.001). Participants initially sensitised to outdoor allergens showed the highest likelihood of developing additional sensitisations (62.3%), compared with pet (56.4%) and indoor allergens (50.9%). In pairwise comparisons, outdoor sensitisation was associated with higher odds of sensitisation expansion compared with indoor (OR 1.60 [95% CI 1.36, 1.88]) and pet sensitisation (OR 1.25 [95% CI 1.03, 1.52]).

**FIGURE 4:**
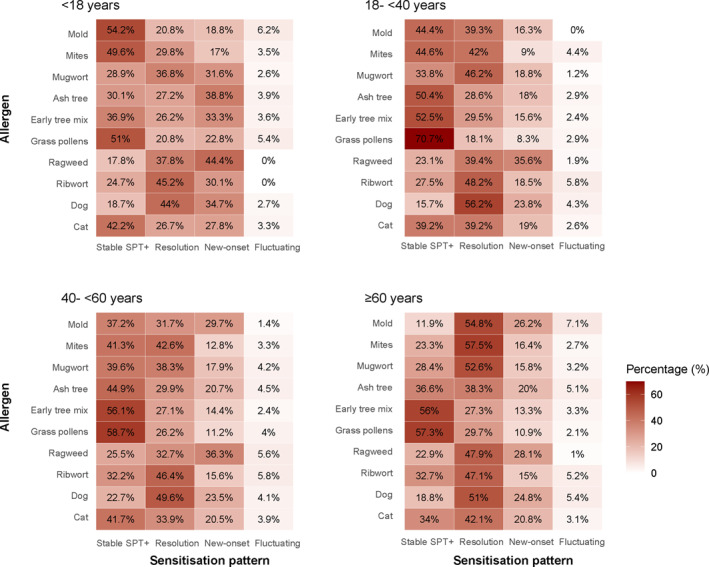
Allergen‐specific patterns by age strata. SPT+, positive skin prick test.

Stable sensitisation was most frequent for seasonal allergens such as grass (> 51.3% of sensitised adults). Early tree pollen mix was common in younger adults but resolved increasingly with age (≥ 60 years; Figures 3 and 4). Ragweed and ribwort sensitisation were most variable, contributing disproportionately to the fluctuating group and ragweed new‐onset sensitisation peaked in adolescents (< 18 years; up to 34%). Mite sensitisation declined across ages while mold remained rare and stable. Cat sensitisation increased progressively in younger adults; dog sensitisation resolved in older adults (up to 56% resolution in ≥ 60 years). Overall, fluctuation was uncommon (< 5% per allergen) < 18 years more often developed ragweed (up to 34%) and early tree pollens sensitisation; ≥ 60 years showed higher resolution (dog, 56% and mold, 36%; Figure 4) 40 ≤ 60 years had highest stable sensitisation to grass and mites (> 50%).

Polysensitisation patterns mirrored these trends (Supporting Information S1: Table S8): stable SPT− participants had no allergens; stable SPT + averaged 2 allergens across visits; and fluctuating individuals had the highest counts (mean 3–4 allergens) and observed greatest variability. New‐onset monosensitisation was more frequent in younger ages (19% increase in allergens in < 18 years vs. 5% in ≥ 60 years), while resolution predominated in older adults. Those with resolution showed declining allergen numbers over time (> 1 allergens in Visit 1 vs. 0 in Visit 3).

## 4 Discussion

Our study offers the first longitudinal population‐based, life‐course perspective on aeroallergen sensitisation across a broad age stratum (6‐82 at baseline) and showed that sensitisation patterns are far more dynamic than traditionally assumed. Although overall SPT positivity remained stable (∼35–40%), individual patterns showed marked heterogeneity. While prior literature has framed sensitisation as a largely fixed trait once established [7, 8, 9], we demonstrate clear evidence of new‐onset, resolution and fluctuating patterns across the lifespan > 15% of participants transitioned between sensitised and nonsensitised status, and these transitions were strongly age dependent. New‐onset predominated in < 18 years, fluctuation in midlife and resolution in older adults, extending prior child‐ or adult‐focused findings [2, 3, 4].

We show that age was the primary determinant of sensitisation patterns, aligned with previous reports of declining new‐onset sensitisation with age [10, 11, 12, 13]. Stable sensitisation was most common in young adults, particularly ragweed and tree pollens, whereas older adults were more likely to be stably nonsensitised or experience resolution. Polysensitisation further showed that stable sensitised participants typically carried approximately two allergens, whereas the fluctuating group bore the heaviest burden, averaging three to four allergens over time across age groups. This is consistent with prior work linking polysensitisation by molecular analysis of specific IgE to more severe and unstable allergic phenotypes [14, 15]. Patients sensitised to > 3 allergens have been reported with more severe rhinitis symptoms (19%) compared to nonsensitised or mono‐/dual‐sensitised individuals (8%–12%) [14]. Additionally, resolution always observed loss of polysensitisation whereas new‐onset was generally monosensitisation. While resolution was most frequently observed in older adults, reduced dermal mast cell function and skin thinning with age may blunt SPT responses, potentially mimicking true desensitisation [16].

Nevertheless, the overall pattern remains striking across all age groups: new‐onset sensitisation was predominantly driven by outdoor allergens, particularly ragweed, and pet dander. Our allergen‐specific analysis further revealed that among individuals already sensitised at baseline, 59% developed at least one additional sensitisation during follow‐up with participants initially sensitised to outdoor sensitisations having the highest likelihood of sensitisation expansion (62.3%), followed by pet (56.4%) and indoor (50.9%) sensitisations. These findings may also reflect that outdoor allergen exposure may drive polysensitisation across the life course. Overall, these SPT patterns aligns with ecological observations linking increasing ragweed spread and pet ownership trends to sensitisation dynamics in European populations [17, 18, 19, 20], suggesting that continued or renewed environmental exposure, and not solely predisposition, contributes to the development of new sensitisation later in life.

Risk factor profiles differed substantially by age. Across all age groups, parental allergy emerged as a robust associated factor of both stable and new‐onset sensitisation. Among participants aged < 18 years, classical atopic markers including male sex, parental allergy, and higher eosinophils, predominated. In adults, behavioural and metabolic factors were influential: cumulative smoking exposure was associated with stable or resolution patterns, and lower adiposity appeared protective, contrasting with literature linking obesity to asthma/allergy risk [21]. This likely reflects differences between short‐term symptom outcomes in prior studies and the life‐course pattern approach used here. This finding was robust, as lower adiposity was also observed in sensitised individuals in cross‐sectional analysis. This may relate to body fat distribution rather than total adiposity: visceral fat drives Th1/Th17 inflammation [22, 23, 24] potentially suppressing Th2‐mediated allergic responses, consistent with discordant BMI‐atopy associations reported previously [25, 26, 27].

In our longitudinal analysis, current and former smokers had lower odds of stable sensitisation, and higher cumulative tobacco exposure was modestly associated with reduced likelihood of persistent sensitisation over time. These findings suggest that smoking may attenuate the persistence of allergic sensitisation rather than prevent its development or promote resolution. This is consistent with mixed evidence: while many cross‐sectional studies report higher total IgE among current smokers [28, 29], longitudinal studies suggest a reduced incidence of new sensitisation among sustained smokers [30, 31]. This may reflects dual pro‐inflammatory and immunosuppressive effects of tobacco, which can alter antigen presentation and Th1/Th2 balance [32, 33]. Hence, the net effects on sensitisation may vary by context and allergen. Moreover, total IgE measures may not reflect local cutaneous responses measured by SPT, particularly for aeroallergens [11, 28]. Overall, our results support a modest but directionally consistent influence on sensitisation trajectories. Further studies on sustained versus changing smoking status, allergen class and smoking exposures are required to determine if this observation reflects a true immunologic effect or residual confounding.

The mismatch between sensitisation, clinical manifestations and use of allergy medication is most notable. Despite clear differences in allergic outcomes across sensitisation patterns, overall use of allergy medication was modest and particularly low in participants < 40 years, where only approximately 10% reported taking antihistamines or leukotriene receptor antagonists. The gap is consistent with previous reports that many sensitised individuals, especially adolescents and young adults, either tolerate symptoms, self‐manage without regular pharmacotherapy or remain undiagnosed in primary care [4, 34]. Discrepancies between sensitisation and clinical symptoms may also be influenced by the use of preventive measures such as allergen avoidance or environmental control [35, 36] Low medication use, particularly in the context of fluctuating and resolving sensitisation, may also partly reflect intermittent or mild symptom profiles typically observed in population‐based cohorts [37, 38, 39, 40]. While our study did not directly compare symptomatic outcomes between poly‐ and monosensitisation, our findings align with prior literature suggesting that symptomatic allergies are more prevalent in individuals with poly‐sensitisation. Asymptomatic sensitisation has been observed to be more common in younger individuals with mono‐sensitisation and less frequent family history [20, 39], reflecting the cumulative effect of multiple allergen exposures over time that could contribute to the development of clinical symptoms in polysensitisation. Future integrating detailed symptom data with longitudinal sensitisation patterns could further elucidate these relationships.

A novel contribution of our study is the identification of a distinct group with fluctuating sensitisation. While this group comprised only 4%–8% of the population, it carried the heaviest allergen burden, had higher adiposity and smoking exposures. Unlike stable sensitisation, which was strongly associated with parental allergy and eosinophils, fluctuation was linked to metabolic, inflammatory, and environmental factors. We lacked quantitative wheal size data and therefore could not formally separate borderline SPT reactions from unequivocal positives in the fluctuation group. Nonetheless, allergen‐specific analyses showed that fluctuation was especially common for seasonal allergens (ragweed, ribwort, tree pollens) and aligned with their respective pollen seasons, especially in adolescents and in young adults. This seasonal and allergen‐specific supports an interpretation that environmental exposure, rather than technical variability alone, contributes to the observed fluctuations. While we cannot exclude that some fluctuations reflect borderline reactivity, the strong alignment with seasonal pollen exposure suggests that at least part of this pattern represent genuine temporal variation in sensitisation. This aligns with recent molecular IgE‐profiling in the LEAD cohort, demonstrating that profiling at the individual allergen level can unmask heterogeneity in sensitisation even in adult asthma patients [41]. This prior study, however, is cross‐sectional and may not directly reflect temporal fluctuations, while the longitudinal SPT findings here demonstrate dynamic changes in sensitisation patterns over time, particularly for seasonal allergens such as ragweed and tree pollens.

Beyond overall temporal trends, qualitative shifts in specific allergen groups may reflect environmental and lifestyle changes. The decline in house dust mite sensitisation may indicate improvements in housing quality, indoor ventilation and humidity control over recent decades, consistent with European population trends showing declining mite exposure alongside better insulation and air quality control [42, 43, 44]. In contrast, decreased animal sensitisation likely reflects increased pet ownership in Austria while the growing prevalence and fluctuation of pollen sensitisation likely results from increasing pollen loads, extended flowering seasons or northward spread driven by climate change, urbanisation and land‐use changes [17, 18, 19, 45, 46]. Grass pollen notably exhibited a gradual rise before reaching plateauing, while trends for other aeroallergens varied by region and year. Other contributors include environmental exposures, including pollutants such as diesel exhaust carbon particles (which are known to bind to major allergen such as Lol p1 in *in vitro* conditions [47]) [48, 49, 50], and geographical location [51]; hormonal shifts, intercurrent illnesses, metabolic changes, diet and activity [1, 36, 52, 53, 54]; and aging‐related changes in skin and immune function [55].

In summary these findings suggest that fluctuation sensitisation is shaped by environmental and systemic factors rather than by classical atopy. Clinically, fluctuating polysensitisation may contribute to variable or seasonal symptoms, often linked to more severe allergic disease and intermittent rhinitis or asthma not captured by single cross‐sectional assessments. Recognition of fluctuation as a standalone phenotype highlights the importance of repeated testing and consideration of tailored interventions, including allergen immunotherapy or exposure‐reduction strategies.

Strengths of this study includes a comprehensive assessment of factors influencing aeroallergen sensitisation in a large, population‐based cohort with repeated and standardised SPT measurements over 12 years and with a broad age stratum. This design enabled us to characterise resolution and fluctuations, which are rarely reported in the literature. Importantly, the study integrated immunological markers, parental allergy history, lifestyle factors including detailed body composition and smoking, and environmental exposures, providing a multidimensional view of associated factors of sensitisation patterns across the life course. Several limitations merit consideration. First, while our 8–12‐year follow‐up reveals age‐dependent sensitisation patterns, individual transitions between age strata were not captured within this timeframe. Second, sensitisation was assessed by SPT only; allergen‐specific IgE was not available for all participants which could have provided complementary information on sensitisation intensity and mechanisms. Third, although we captured several host, metabolic, and environmental risk factors, data on key contributors to sensitisation dynamics, such as hormonal changes, illness history, diet, and detailed pollutant exposure, were not available, limiting our ability to fully characterise the drivers of fluctuation and resolution. Fourth, as the cohort is Austrian and largely European, results may not generalise to populations with different genetic backgrounds, allergen exposures or lifestyles. Finally, our findings should be interpreted in consideration of several methodological limitations. Although SPT visits were not standardised to a specific season or month, the distribution of visit timing across the three assessments was comparable, reducing the likelihood of seasonal bias. Additionally, no explicit restrictions were placed on alcohol intake or vigorous exercise, which can transiently influence skin perfusion. While batch‐to‐batch variability in allergen vials cannot be entirely excluded, our control charts demonstrated > 98% validity for histamine/saline controls suggesting minimal technical bias, and operator consistency was maintained through trained technicians. Future studies involving SPT could use automated SPT devices to further standardise testing [56].

## 5 Conclusion

In conclusion, our study demonstrates that allergic sensitisation is a dynamic trait rather than a fixed lifelong characteristic. Resolution and fluctuation occurred across the life course, and new‐onset sensitisation can develop well into adolescence and adulthood. These findings challenge long‐standing assumptions in allergy studies and highlight the importance of longitudinal monitoring. Recognising these patterns can guide age‐ and risk‐specific approaches aimed at preventing the progression to persistent allergic disease.

## Author Contributions

All authors have read and given their final approval for this version to be published. C.J.M.L was involved in the conceptualisation, methodology, formal analysis, interpretation of the data and the writing and editing of the manuscript. A.O. was involved in the formal analysis and interpretation of the data in the manuscript. S.P. was involved in the final review of the manuscript. R.B.‐K., M.‐K.B., F.M.E.F., E.F.M.W. were involved in supervision, and the final review and editing of the manuscript.

## Funding

The Austrian LEAD Study Austrian LEAD study has unrestricted financial support from the Ludwig Boltzmann Gesellschaft, Sigmund Freud Private University Faculty of Medicine, Vienna Healthcare Group, the Lower Austrian Health and Social Fund, AstraZeneca, Chiesi Farmaceutici, GlaxoSmithKline, and Menarini Pharma. None of the supporting parties has any participation in the data, nor have they contributed to the design or the content of the manuscript.

## Ethics Statement

The study was conducted within the framework of the LEAD study which has been approved by the local Ethics committee of Vienna (EK‐11‐117–0711) with the project identification code, NCT017257518 (ClinicalTrials.gov) and in accordance with the Declaration of Helsinki.

## Conflicts of Interest

The authors declare no conflicts of interest. R.B.‐K. and M.‐K.B. reports consulting fees from AstraZeneca, Menarini, Sanofi, Boehringer Ingelheim, Novartis Pharma, GSK and Sanofi for advisory board members outside the submitted work. F.M.E.F. reports consulting fees from MSD and Sanofi and honoraria from AstraZeneca, Chiesi, GSK, Sanofi and Pfizer outside of the submitted work. C.J.M.L, A.O., S.P., E.F.M.W. have nothing to disclose.

## Supporting information


Supporting Information S1

## Data Availability

Data relevant to this study can be made available upon request to the corresponding author.
