# Supplementary material for: Temporal Patterns of Allergen Sensitisation in the General Population: The LEAD Study
Source: Clin Transl Allergy. 2026 Jun 12;16(6):e70181. doi: 10.1002/clt2.70181 (PMC13263163; doi:10.1002/clt2.70181)
Supplement: Supplementary file 1 — Supporting Information S1 [file CLT2-16-e70181-s001.docx]

**Supplementary materials**

**Temporal patterns of allergen sensitisation in the general population: the LEAD study**

**Author list and affiliations**

Charmaine J. M. Lim ^1,2^, Abdelrahman Omar ^2^, Severin Pötscher ^3^, Marie-Kathrin Breyer ^1,4^, Frits M. E. Franssen ^5,6^, Emiel F. M. Wouters ^1,2,5*^, Robab Breyer-Kohansal ^1,3*^

^1^Ludwig Boltzmann Institute for Lung Health, Vienna, Austria

^2^Sigmund Freud Private University, Faculty of Medicine, Vienna, Austria

^3^Department of Respiratory and Pulmonary Diseases, Clinic Hietzing, Vienna Healthcare Group, Vienna, Austria

^4^Department of Respiratory and Pulmonary Diseases, Site Penzing of Clinic Ottakring, Vienna Healthcare Group, Vienna, Austria.

^5^NUTRIM, Institute for Nutrition and Translational Research in Metabolism, Maastricht, Netherlands

^6^Department of Respiratory Medicine, Maastricht University Medical Center, Maastricht, the Netherlands

*Authors contributed equally to this work as last authors

**Supplementary table 1**. Baseline characteristics of sensitisation patterns in individuals aged <18 years.

|  | **Stable non-sensitised** | **Stable sensitised** | **Resolution** | **New-onset** | **Fluctuating** | **p-value** |
| --- | --- | --- | --- | --- | --- | --- |
| Total N, % | 226 (50.1) | 147 (32.6) | 11 (2.4) | 44 (9.8) | 23 (5.1) |  |
| Males, % | 99 (43.8) | 83 (56.5) | 4 (36.4) | 20 (45.5) | 10 (43.5) | 0.152 |
| Age | 12.3 ± 3.3 | 12.4 ± 3.3 | 14.0 ± 2.8 | 11.3 ± 3.3 | 12.5 ± 3.3 | 0.133 |
| **Body composition** | |  |  |  |  |  |
| Waist-height, ratio | 0.5 ± 0.1 | 0.5 ± 0.1 | 0.5 ± 0.1 | 0.5 ± 0.0 | 0.5 ± 0.1 | 0.491 |
| BMI, kg/m^2^ | 18.7 ± 3.4 | 19.0 ± 3.5 | 20.8 ± 2.9* | 17.7 ± 3.0* | 19.6 ± 3.1 | 0.030 |
| FMI, kg/m^2^ | 5.4 ± 2.3 | 5.5 ± 2.4 | 7.0 ± 2.3 | 5.1 ± 1.7 | 5.7 ± 2.6 | 0.170 |
| LMI, kg/m^2^ | 12.7 ± 1.9 | 13.0 ± 2.3 | 13.2 ± 1.6 | 12.2 ± 1.6 | 13.6 ± 2.2 | 0.057 |
| **Biomarkers** |  |  |  |  |  |  |
| hsCRP, mg/dL | 0.9 ± 3.0 | 1.0 ± 2.8 | 1.6 ± 2.2 | 0.5 ± 0.9 | 1.1 ± 2.3 | 0.787 |
| Neutrophils, cells/L | 3.7 ± 1.4 | 3.6 ± 1.5 | 4.1 ± 1.3 | 3.4 ± 1.2 | 4.0 ± 1.3 | 0.400 |
| Eosinophils, cells/µL | 167.8 ± 162.8 | 265.2 ± 196.2* | 137.3 ± 56.4 | 195.3 ± 156.6 | 152.5 ± 129.9 | <0.001 |
| **Medication and clinical presentation** | |  |  |  |  |  |
| Use of any allergy medication, % | 2 (0.9) | 13 (8.8)* | 0 (0.0) | 0 (0.0) | 2 (8.7)* | 0.001 |
| Sinonasal disease, % | 9 (4.0) | 3 (2.0) | 0 (0.0) | 0 (0.0) | 1 (4.3) | 0.532 |
| Allergy, % | 8 (3.5) | 62 (42.2)* | 1 (9.1) | 3 (7.0) | 4 (17.4)* | <0.001 |
| Rhino-conjunctivitis, % | 5 (2.2) | 64 (43.5)* | 0 (0.0) | 0 (0.0) | 3 (13.0) | <0.001 |
| Eczema, % | 18 (8.0) | 22 (16.9) | 0 (0.0) | 2 (4.5) | 3 (14.3) | 0.033 |
| Nocturnal symptoms, % | 6 (66.7) | 7 (63.6) | 2 (100.0) | 1 (100.0) | 1 (100.0) | 0.736 |
| Chronic cough, % | 6 (2.7) | 2 (1.4) | 0 (0.0) | 1 (2.3) | 0 (0.0) | 0.822 |
| Wheeze, % | 9 (4.0) | 13 (8.8) | 1 (9.1) | 0 (0.0) | 1 (4.3) | 0.123 |
| Asthma, % | 3 (1.3) | 13 (8.8)* | 0 (0.0) | 0 (0.0) | 2 (8.7) | 0.002 |
| **Lifestyle and early-life events** | | |  |  |  |  |
| Never smokers, % | 218 (96.5) | 144 (98.0) | 9 (81.8) | 42 (100.0) | 23 (100.0) | 0.019 |
| Current smokers, % | 8 (3.5) | 3 (2.0) | 2 (18.2) | 0 (0.0) | 0 (0.0) |  |
| Pack-years | 0.0 ± 0.2 | 0.0 ± 0.0 | 0.0 ± 0.1 | 0.0 ± 0.0 | 0.0 ± 0.0 | 0.372 |
| Passive smoking, % | 135 (59.7) | 78 (53.1) | 7 (63.6) | 22 (50.0) | 12 (52.2) | 0.588 |
| Urbanicity, % | 198 (87.6) | 120 (81.6) | 10 (90.9) | 39 (88.6) | 22 (95.7) | 0.275 |
| Residence near main road, % | 127 (57.0) | 80 (55.2) | 6 (54.5) | 24 (55.8) | 14 (60.9) | 0.988 |
| Exposure to dust, % | 4 (3.6) | 0 (0.0) | 0 (0.0) | 1 (6.2) | 0 (0.0) | 0.338 |
| Socioeconomic status, score | 5.7 ± 2.8 | 5.7 ± 2.8 | 4.8 ± 2.6 | 6.6 ± 3.3 | 5.9 ± 2.6 | 0.281 |
| Socioeconomic status, low, % | 165 (73.0) | 106 (72.1) | 9 (81.8) | 26 (59.1) | 17 (73.9) | 0.373 |
| PM_10_, µg/m^3^ | 20.8 ± 1.4 | 20.8 ± 1.3 | 20.7 ± 0.8 | 20.7 ± 1.6 | 20.9 ± 1.2 | 0.991 |
| NO_2_, µg/m^3^ | 20.3 ± 3.7 | 21.0 ± 4.0 | 20.5 ± 3.7 | 20.6 ± 4.5 | 20.0 ± 3.6 | 0.572 |
| Parental allergies, % | 104 (46.6) | 100 (69.9)* | 6 (54.5) | 26 (60.5) | 11 (50.0) | 0.001 |
| **Longitudinal analysis- incident conditions/changes in states** | | | | | |  |
| New exposures, % | 76 (57.6) | 56 (58.3) | 8 (80.0) | 13 (59.1) | 9 (60.0) | 0.744 |
| Smoking, quitters, % | 2 (0.9) | 1 (0.7) | 1 (9.1) | 0 (0.0) | 0 (0.0) | 0.060 |
| Smoking, persistent, % | 6 (2.7) | 2 (1.4) | 1 (9.1) | 0 (0.0) | 0 (0.0) | 0.287 |
| ∆ BMI, kg/m^2^.year^-1^ | 0.5 ± 0.4 | 0.5 ± 0.4 | 0.6 ± 0.5 | 0.5 ± 0.4 | 0.6 ± 0.5 | 0.849 |
| ∆ FMI, kg/m^2^.year^-1^ | 0.2 ± 0.3 | 0.2 ± 0.3 | 0.3 ± 0.4 | 0.2 ± 0.2 | 0.2 ± 0.3 | 0.703 |
| ∆ Eosinophils, cells/µL.year^-1^ | -5.4 ± 20.0 | -6.4 ± 23.9 | -2.4 ± 9.6 | -0.4 ± 25.0 | -1.0 ± 15.8 | 0.504 |
| ∆ IgE, U/ml.year^-1^ | -6.7 ±39.4 | 1.5 ±130.6 | -0.6 ±33.8 | 78.9 ±700.5 | -11.7 ±35.0 | 0.302 |
| Data is presented as mean ± standard deviations or frequency (%). *Significance is considered where p<0.05 for comparisons with the stable non-sensitised group. BMI, body mass index; FMI, fat mass index; LMI, lean mass index; hsCRP, high-sensitivity C-reactive protein; IgE, immunoglobulin E; NO_2_, nitrogen dioxide. ∆ is calculated as (values in Visit 3 minus values in Visit 1)/total visit interval. ∆ IgE is calculated by (values in Visit 3 minus values in Visit 1)/visit intervals between Visits 3 and 2. Significance is considered where p<0.05. | | | | | | |

**Supplementary table 2**. Baseline characteristics of sensitisation patterns in individuals aged 18-<40 years.

|  | **Stable non-sensitised** | **Stable sensitised** | **Resolution** | **New-onset** | **Fluctuating** | **p-value** |
| --- | --- | --- | --- | --- | --- | --- |
| Total N, % | 535 (45.4) | 465 (39.4) | 65 (5.5) | 64 (5.4) | 50 (4.2) |  |
| Males, % | 225 (42.1) | 298 (64.1)* | 33 (50.8) | 29 (45.3) | 25 (50.0) | <0.001 |
| Age | 30.4 ± 6.2 | 30.0 ± 6.3 | 31.1 ± 5.9 | 31.3 ± 5.5 | 30.7 ± 6.3 | 0.415 |
| **Body composition** | |  |  |  |  |  |
| Waist-height, ratio | 0.5 ± 0.1 | 0.5 ± 0.1 | 0.5 ± 0.1 | 0.5 ± 0.0 | 0.5 ± 0.1 | 0.411 |
| BMI, kg/m^2^ | 24.0 ± 4.5 | 23.7 ± 4.0 | 23.9 ± 3.5 | 23.1 ± 2.9 | 24.0 ± 4.1 | 0.576 |
| FMI, kg/m^2^ | 7.3 ± 3.1 | 6.7 ± 2.9* | 7.3 ± 2.8 | 6.6 ± 2.2* | 7.0 ± 2.6 | 0.018 |
| LMI, kg/m^2^ | 15.7 ± 2.3 | 16.2 ± 2.3* | 15.8 ± 2.3 | 15.6 ± 2.2 | 15.8 ± 2.4 | 0.061 |
| **Biomarkers** | |  |  |  |  |  |
| hsCRP, mg/dL | 1.7 ± 3.3 | 1.5 ± 3.9 | 1.8 ± 2.6 | 1.1 ± 1.4 | 1.3 ± 1.7 | 0.571 |
| Neutrophils, cells/L | 3.9 ± 1.5 | 3.8 ± 1.6 | 3.7 ± 1.3 | 3.8 ± 1.6 | 3.9 ± 1.5 | 0.559 |
| Eosinophils, cells/µL | 140.8 ± 128.3 | 179.8 ± 152.9* | 147.6 ± 94.6 | 121.2 ± 74.7 | 126.9 ± 94.1 | <0.001 |
| **Medication and clinical presentation** | |  |  |  |  |  |
| Use of any allergy medication, % | 1 (0.2) | 44 (9.5) | 1 (1.5) | 0 (0.0) | 2 (4.0) | <0.001 |
| Sinonasal disease, % | 13 (2.4) | 10 (2.2) | 2 (3.1) | 1 (1.6) | 0 (0.0) | 0.807 |
| Allergy, % | 57 (10.7) | 299 (64.3)* | 22 (33.8)* | 11 (17.2) | 9 (18.4) | <0.001 |
| Rhino-conjunctivitis, % | 22 (4.1) | 302 (64.9)* | 19 (29.2)* | 12 (18.8)* | 10 (20.0)* | <0.001 |
| Eczema, % | 53 (10.0) | 103 (25.6)* | 11 (18.0) | 7 (11.3) | 4 (8.7) | <0.001 |
| Nocturnal symptoms, % | 21 (70.0) | 18 (45.0) | 4 (100.0) | 0 (0.0) | 1 (25.0) | 0.008 |
| Chronic cough, % | 22 (4.1) | 29 (6.2) | 1 (1.5) | 6 (9.4) | 2 (4.0) | 0.163 |
| Wheeze, % | 36 (6.7) | 50 (10.8) | 4 (6.2) | 5 (7.8) | 5 (10.0) | 0.211 |
| Asthma, % | 12 (2.2) | 69 (14.8)* | 5 (7.7) | 3 (4.7) | 4 (8.0) | <0.001 |
| **Lifestyle and early-life events** | | |  |  |  |  |
| Never smokers, % | 270 (50.5) | 249 (53.5) | 30 (46.2) | 35 (54.7) | 29 (58.0) | 0.905 |
| Former smokers, % | 121 (22.6) | 97 (20.9) | 17 (26.2) | 14 (21.9) | 8 (16.0) |  |
| Current smokers, % | 144 (26.9) | 119 (25.6) | 18 (27.7) | 15 (23.4) | 13 (26.0) |  |
| Pack-years | 3.7 ± 6.8 | 2.6 ± 5.0 | 3.2 ± 5.4 | 3.1 ± 5.7 | 4.0 ± 7.3 | 0.051 |
| Passive smoking, % | 363 (67.9) | 303 (65.2) | 46 (70.8) | 46 (71.9) | 32 (64.0) | 0.700 |
| Urbanicity, % | 468 (87.5) | 418 (89.9) | 58 (89.2) | 60 (93.8) | 46 (92.0) | 0.463 |
| Residence near main road, % | 327 (61.7) | 315 (67.9) | 45 (69.2) | 38 (60.3) | 38 (77.6) | 0.067 |
| Exposure to dust, % | 91 (17.0) | 73 (15.7) | 13 (20.0) | 14 (21.9) | 9 (18.0) | 0.720 |
| Socioeconomic status, score | 12.4 ± 3.8 | 12.2 ± 4.1 | 12.6 ± 3.8 | 12.2 ± 4.0 | 12.1 ± 4.0 | 0.810 |
| Socioeconomic status, low, % | 92 (17.2) | 105 (22.6) | 10 (15.4) | 11 (17.2) | 12 (24.0) | 0.186 |
| PM_10_, µg/m^3^ | 21.1 ± 1.2 | 21.1 ± 1.3 | 21.3 ± 1.5 | 21.1 ± 1.3 | 21.3 ± 1.2 | 0.652 |
| NO_2_, µg/m^3^ | 20.7 ± 4.1 | 20.7 ± 4.0 | 21.7 ± 5.2 | 21.6 ± 5.1 | 21.1 ± 4.5 | 0.276 |
| Parental allergies, % | 124 (23.9) | 192 (43.7)* | 17 (27.0) | 16 (26.2) | 13 (27.7) | <0.001 |
| **Longitudinal analysis- incident conditions/changes in states** | | | | | |  |
| New exposures, % | 181 (35.8) | 137 (30.6) | 23 (36.5) | 18 (30.0) | 19 (38.8) | 0.396 |
| Smoking, quitters, % | 48 (9.2) | 42 (9.3) | 4 (6.6) | 3 (4.8) | 5 (10.0) | 0.751 |
| Smoking, persistent, % | 71 (13.4) | 55 (12.0) | 9 (14.1) | 9 (14.3) | 8 (16.0) | 0.904 |
| ∆ BMI, kg/m^2^.year^-1^ | 0.1 ± 0.3 | 0.2 ± 0.3 | 0.1 ± 0.3 | 0.1 ± 0.2 | 0.2 ± 0.2 | 0.852 |
| ∆ FMI, kg/m^2^.year^-1^ | 0.1 ± 0.2 | 0.1 ± 0.2 | 0.1 ± 0.2 | 0.1 ± 0.2 | 0.1 ± 0.2 | 0.544 |
| ∆ Eosinophils, cells/µL.year^-1^ | 0.8 ± 14.0 | -0.4 ± 17.0 | 1.9 ± 13.6 | 1.5 ± 10.1 | 0.7 ± 8.4 | 0.625 |
| ∆ IgE, U/ml.year^-1^ | -0.3 ± 20.7 | -2.1 ± 40.8 | -1.2 ± 10.0 | -3.3 ± 15.3 | -3.7 ± 15.1 | 0.834 |
| Data is presented as mean ± standard deviations or frequency (%). *Significance is considered where p<0.05 for comparisons with the stable non-sensitised group. BMI, body mass index; FMI, fat mass index; LMI, lean mass index; hsCRP, high-sensitivity C-reactive protein; IgE, immunoglobulin E; NO_2_, nitrogen dioxide. ∆ is calculated as (values in Visit 3 minus values in Visit 1)/total visit interval. ∆ IgE is calculated by (values in Visit 3 minus values in Visit 1)/visit intervals between Visits 3 and 2. Significance is considered where p<0.05. | | | | | | |

**Supplementary table 3**. Baseline characteristics of sensitisation patterns in individuals aged 40-<60 years.

|  | **Stable non-sensitised** | **Stable sensitised** | **Resolution** | **New-onset** | **Fluctuating** | **p-value** |
| --- | --- | --- | --- | --- | --- | --- |
| Total N, % | 1151 (53.3) | 696 (32.3) | 120 (5.6) | 95 (4.4) | 96 (4.4) |  |
| Males, % | 511 (44.4) | 381 (54.7)* | 61 (50.8) | 45 (47.4) | 46 (47.9) | 0.001 |
| Age | 50.6 ± 5.5 | 49.4 ± 5.3* | 50.6 ± 5.6 | 49.8 ± 5.8 | 51.0 ± 5.3 | <0.001 |
| **Body composition** | |  |  |  |  |  |
| Waist-height, ratio | 0.6 ± 0.1 | 0.5 ± 0.1* | 0.6 ± 0.1 | 0.5 ± 0.1 | 0.6 ± 0.1* | <0.001 |
| BMI, kg/m^2^ | 26.2 ± 4.5 | 25.7 ± 4.1* | 26.8 ± 4.8 | 25.8 ± 4.2 | 27.6 ± 6.2* | 0.001 |
| FMI, kg/m^2^ | 9.0 ± 3.4 | 8.3 ± 3.0* | 9.0 ± 3.0 | 8.7 ± 3.1 | 9.7 ± 4.1 | <0.001 |
| LMI, kg/m^2^ | 16.3 ± 2.2 | 16.5 ± 2.2 | 16.7 ± 2.5 | 16.4 ± 2.4 | 16.8 ± 2.6 | 0.091 |
| **Biomarkers** | |  |  |  |  |  |
| hsCRP, mg/dL | 2.0 ± 2.9 | 1.8 ± 2.8 | 1.9 ± 2.2 | 1.7 ± 1.7 | 2.2 ± 5.3 | 0.549 |
| Neutrophils, cells/L | 4.0 ± 1.4 | 3.8 ± 1.5 | 3.9 ± 1.3 | 4.0 ± 1.4 | 4.1 ± 1.4 | 0.063 |
| Eosinophils, cells/µL | 145.5 ± 106.0 | 173.3 ± 141.9* | 166.8 ± 145.1 | 154.6 ± 115.3 | 161.7 ± 131.5 | <0.001 |
| **Medication and clinical presentations** | |  |  |  |  |  |
| Use of any allergy medication, % | 16 (1.4) | 46 (6.6)* | 2 (1.7) | 4 (4.2) | 0 (0.0) | <0.001 |
| Sinonasal disease, % | 13 (2.4) | 10 (2.2) | 2 (3.1) | 1 (1.6) | 0 (0.0) | 0.807 |
| Allergy, % | 57 (14.7) | 299 (68.0)* | 22 (39.2)* | 11 (23.2)* | 9 (26.0)* | <0.001 |
| Rhino-conjunctivitis, % | 22 (5.6) | 302 (66.5)* | 19 (28.3)* | 12 (13.7) | 10 (13.5) | <0.001 |
| Eczema, % | 53 (10.0) | 103 (25.6)* | 11 (18.0) | 7 (11.3) | 4 (8.7) | <0.001 |
| Nocturnal symptoms, % | 21 (70.0) | 18 (45.0) | 4 (100.0) | 0 (0.0) | 1 (25.0) | 0.785 |
| Chronic cough, % | 22 (4.1) | 29 (6.2) | 1 (1.5) | 6 (9.4) | 2 (4.0) | 0.163 |
| Wheeze, % | 36 (6.7) | 50 (10.8) | 4 (6.2) | 5 (7.8) | 5 (10.0) | 0.211 |
| Asthma, % | 12 (3.4) | 69 (15.1)* | 5 (7.5) | 3 (3.2) | 4 (6.2) | <0.001 |
| **Lifestyle and early-life events** | | |  |  |  |  |
| Never smokers, % | 421 (36.6) | 315 (45.3)* | 55 (45.8) | 45 (47.4) | 37 (38.5) | 0.007 |
| Former smokers, % | 427 (37.1) | 241 (34.6) | 36 (30.0) | 28 (29.5) | 38 (39.6) |  |
| Current smokers, % | 303 (26.3) | 140 (20.1)* | 29 (24.2) | 22 (23.2) | 21 (21.9) |  |
| Pack-years | 12.5 ± 18.1 | 9.0 ± 15.5 | 11.4 ± 14.9 | 9.8 ± 14.1 | 13.3 ± 17.0 | <0.001 |
| Passive smoking, % | 758 (65.9) | 441 (63.4) | 81 (67.5) | 63 (66.3) | 67 (69.8) | 0.651 |
| Urbanicity, % | 949 (82.5) | 559 (80.3) | 89 (74.2)* | 78 (82.1) | 70 (72.9)* | 0.050 |
| Residence near main road, % | 668 (58.8) | 392 (56.6) | 61 (50.8) | 48 (51.1) | 58 (60.4) | 0.275 |
| Exposure to dust, % | 254 (22.1) | 146 (21.0) | 31 (26.1) | 22 (23.2) | 29 (30.2) | 0.275 |
| Socioeconomic status, score | 12.8 ± 3.6 | 14.0 ± 3.4* | 12.7 ± 3.5 | 12.5 ± 3.6 | 12.9 ± 3.6 | <0.001 |
| Socioeconomic status, low, % | 164 (14.2) | 55 (7.9)* | 12 (10.0) | 12 (12.6) | 10 (10.4) | 0.002 |
| PM_10_, µg/m^3^ | 20.9 ± 1.2 | 20.9 ± 1.2 | 20.8 ± 1.2 | 20.7 ± 1.0 | 21.2 ± 1.2 | 0.161 |
| NO2, µg/m^3^ | 20.9 ± 4.1 | 21.2 ± 4.5 | 21.1 ± 3.8 | 20.5 ± 3.3 | 20.1 ± 3.5 | 0.160 |
| Parental allergies, % | 117 (10.7) | 121 (18.8)* | 16 (14.0) | 11 (12.0) | 15 (17.4) | <0.001 |
| **Longitudinal analysis- incident conditions/changes in states** | | | | | |  |
| New exposures, % | 328 (29.5) | 178 (26.4) | 29 (25.0) | 33 (35.5) | 29 (30.9) | 0.261 |
| Smoking, quitters, % | 99 (8.7) | 47 (6.9) | 8 (6.7) | 3 (3.2) | 9 (9.4) | 0.234 |
| Smoking, persistent, % | 169 (14.9) | 68 (9.9)* | 18 (15.1) | 14 (15.1) | 11 (11.5) | 0.039 |
| ∆ BMI, kg/m^2^.year^-1^ | 0.1 ± 0.2 | 0.1 ± 0.2 | 0.1 ± 0.2 | 0.1 ± 0.3 | 0.1 ± 0.3 | 0.447 |
| ∆ FMI, kg/m^2^.year^-1^ | 0.1 ± 0.2 | 0.1 ± 0.2 | 0.1 ± 0.2 | 0.1 ± 0.2 | 0.1 ± 0.2 | 0.588 |
| ∆ Eosinophils, cells/µL.year^-1^ | 1.9 ± 21.0 | -0.6 ± 16.0 | 0.6 ± 12.8 | 0.7 ± 11.2 | 0.3 ± 14.4 | 0.085 |
| ∆ IgE, U/ml.year^-1^ | 0.5 ± 18.5 | -2.6 ± 61.5 | 5.9 ± 90.9 | 0.1 ± 20.7 | -5.1 ± 23.3 | 0.264 |
| Data is presented as mean ± standard deviations or frequency (%). *Significance is considered where p<0.05 for comparisons with the stable non-sensitised group. BMI, body mass index; FMI, fat mass index; LMI, lean mass index; hsCRP, high-sensitivity C-reactive protein; IgE, immunoglobulin E; NO_2_, nitrogen dioxide. ∆ is calculated as (values in Visit 3 minus values in Visit 1)/total visit interval. ∆ IgE is calculated by (values in Visit 3 minus values in Visit 1)/visit intervals between Visits 3 and 2. Significance is considered where p<0.05. | | | | | | |

**Supplementary table 4**. Baseline characteristics of sensitisation patterns in individuals aged ≥60 years.

|  | **Stable non-sensitised** | **Stable sensitised** | **Resolution** | **New-onset** | **Fluctuating** | **p-value** |
| --- | --- | --- | --- | --- | --- | --- |
| Total N, % | 832 (66.1) | 238 (18.9) | 85 (6.8) | 40 (3.2) | 63 (5.0) |  |
| Males, % | 396 (47.6) | 129 (54.2) | 39 (45.9) | 18 (45.0) | 37 (58.7) | 0.186 |
| Age | 67.7 ± 4.9 | 66.5 ± 4.5* | 67.6 ± 5.3 | 66.4 ± 4.7 | 67.8 ± 4.9 | 0.008 |
| **Body composition** | |  |  |  |  |  |
| Waist-height, ratio | 0.6 ± 0.1 | 0.6 ± 0.1 | 0.6 ± 0.1 | 0.6 ± 0.1 | 0.6 ± 0.1* | 0.003 |
| BMI, kg/m^2^ | 27.3 ± 4.1 | 26.9 ± 3.9 | 27.2 ± 4.3 | 27.3 ± 4.4 | 29.0 ± 5.6* | 0.011 |
| FMI, kg/m^2^ | 10.0 ± 3.3 | 9.6 ± 3.0 | 10.1 ± 3.6 | 10.2 ± 3.4 | 10.7 ± 3.9 | 0.139 |
| LMI, kg/m^2^ | 16.4 ± 2.1 | 16.4 ± 2.1 | 16.2 ± 1.9 | 16.4 ± 2.3 | 17.2 ± 2.6 | 0.086 |
| **Biomarkers** | |  |  |  |  |  |
| hsCRP, mg/dL | 2.2 ± 2.7 | 2.0 ± 2.4 | 3.6 ± 8.3 | 2.7 ± 3.1 | 3.0 ± 3.7 | 0.002 |
| Neutrophils, cells/L | 3.9 ± 1.3 | 3.8 ± 1.1 | 4.0 ± 1.4 | 3.9 ± 1.1 | 4.2 ± 1.3 | 0.114 |
| Eosinophils, cells/µL | 148.5 ± 113.7 | 195.9 ± 175.7* | 174.3 ± 191.2 | 130.0 ± 94.8 | 163.8 ± 121.0 | <0.001 |
| **Medication and clinical presentations** | |  |  |  |  |  |
| Use of any allergy medication, % | 18 (2.2) | 12 (5.0) | 1 (1.2) | 0 (0.0) | 1 (1.6) | 0.078 |
| Sinonasal disease, % | 33 (4.0) | 14 (5.9) | 2 (2.4) | 1 (2.5) | 2 (3.2) | 0.559 |
| Allergy, % | 116 (13.9) | 137 (57.6)* | 22 (25.9)* | 12 (30.0)* | 16 (25.4)* | <0.001 |
| Rhino-conjunctivitis, % | 34 (4.1) | 133 (55.9)* | 13 (15.3)* | 7 (17.5)* | 12 (19.0)* | <0.001 |
| Eczema, % | 104 (12.6) | 47 (23.0)* | 15 (17.9) | 6 (15.0) | 7 (11.7) | 0.004 |
| Nocturnal symptoms, % | 32 (3.8) | 14 (5.9) | 2 (2.4) | 1 (2.5) | 5 (7.9) | 0.288 |
| Chronic cough, % | 73 (8.8) | 18 (7.6) | 7 (8.2) | 0 (0.0) | 7 (11.1) | 0.324 |
| Wheeze, % | 64 (7.7) | 25 (10.5) | 5 (5.9) | 4 (10.0) | 7 (11.1) | 0.499 |
| Asthma, % | 25 (3.0) | 23 (9.7)* | 6 (7.1) | 2 (5.0) | 3 (4.8) | <0.001 |
| **Lifestyle and early-life events** | | |  |  |  |  |
| Never smokers, % | 374 (45.0) | 120 (50.4) | 36 (42.4) | 14 (35.0) | 23 (36.5) | 0.014 |
| Former smokers, % | 341 (41.0) | 102 (42.9) | 41 (48.2) | 24 (60.0) | 30 (47.6) |  |
| Current smokers, % | 117 (14.1) | 16 (6.7)* | 8 (9.4) | 2 (5.0) | 10 (15.9) |  |
| Pack-years | 13.5 ± 21.6 | 9.7 ± 16.9* | 10.8 ± 14.9 | 16.9 ± 26.7 | 18.8 ± 28.5 | 0.009 |
| Passive smoking, % | 528 (63.5) | 155 (65.1) | 49 (57.6) | 26 (65.0) | 41 (65.1) | 0.802 |
| Urbanicity, % | 636 (76.4) | 187 (78.6) | 68 (80.0) | 32 (80.0) | 50 (79.4) | 0.871 |
| Residence near main road, % | 455 (55.4) | 115 (48.7) | 53 (63.1) | 22 (57.9) | 37 (59.7) | 0.152 |
| Exposure to dust, % | 174 (20.9) | 53 (22.3) | 21 (24.7) | 12 (30.0) | 18 (28.6) | 0.406 |
| Socioeconomic status, score | 12.3 ± 3.0 | 12.6 ± 3.2 | 12.0 ± 2.8 | 11.6 ± 3.6 | 11.8 ± 2.5 | 0.126 |
| Socioeconomic status, low, % | 94 (11.3) | 28 (11.8) | 10 (11.8) | 9 (22.5) | 8 (12.7) | 0.327 |
| PM_10_, µg/m^3^ | 20.7 ± 1.1 | 20.6 ± 1.0 | 20.8 ± 1.0 | 20.5 ± 0.9 | 21.1 ± 1.3* | 0.010 |
| NO_2_, µg/m^3^ | 21.1 ± 4.2 | 21.0 ± 4.1 | 22.6 ± 4.6* | 20.7 ± 3.1 | 22.2 ± 5.2 | 0.020 |
| Parental allergies, % | 32 (4.2) | 14 (6.7) | 5 (6.4) | 3 (7.7) | 3 (5.2) | 0.528 |
| **Longitudinal analysis- incident conditions/changes in states** | | | | | |  |
| New exposures, % | 235 (29.7) | 66 (28.9) | 25 (30.1) | 12 (32.4) | 11 (18.6) | 0.476 |
| Smoking, quitters, % | 42 (5.1) | 8 (3.4) | 2 (2.4) | 1 (2.5) | 2 (3.3) | 0.568 |
| Smoking, persistent, % | 57 (6.9) | 6 (2.5) | 6 (7.1) | 1 (2.5) | 6 (9.7) | 0.071 |
| ∆ BMI, kg/m^2^.year^-1^ | -0.0 ± 0.2 | -0.0 ± 0.2 | -0.0 ± 0.2 | 0.0 ± 0.3 | -0.0 ± 0.2 | 0.566 |
| ∆ FMI, kg/m^2^.year^-1^ | 0.0 ± 0.2 | 0.0 ± 0.2 | 0.0 ± 0.2 | 0.0 ± 0.2 | 0.0 ± 0.2 | 0.811 |
| ∆ Eosinophils, cells/µL.year^-1^ | 1.4 ± 13.0 | 0.6 ± 16.0 | 2.3 ± 15.6 | 7.0 ± 10.9 | 1.1 ± 12.0 | 0.098 |
| ∆ IgE, U/ml.year^-1^ | 0.4 ± 39.2 | 3.4 ± 51.6 | 7.2 ± 62.8 | 14.1 ± 81.1 | 22.2 ± 176.1 | 0.072 |
| Data is presented as mean ± standard deviations or frequency (%). *Significance is considered where p<0.05 for comparisons with the stable non-sensitised group. BMI, body mass index; FMI, fat mass index; LMI, lean mass index; hsCRP, high-sensitivity C-reactive protein; IgE, immunoglobulin E; NO_2_, nitrogen dioxide. ∆ is calculated as (values in Visit 3 minus values in Visit 1)/total visit interval. ∆ IgE is calculated by (values in Visit 3 minus values in Visit 1)/visit intervals between Visits 3 and 2. Significance is considered where p<0.05. | | | | | | |

**Supplementary table 5.** Latent class membership probabilities for sensitisation trajectories.

| **Class** | **Stable non-sensitised** | **Stable sensitised** | **Resolution** | **New-onset** | **Fluctuating** |
| --- | --- | --- | --- | --- | --- |
| Total population |  |  |  |  |  |
| 1 | 0 | 82.7 | 9.4 | 6.7 | 2.1 |
| 2 | 86.4 | 0 | 3.9 | 3.7 | 6 |
| <18 years |  |  |  |  |  |
| 1 | 0 | 80.3 | 2.7 | 14.8 | 2.2 |
| 2 | 84.3 | 0 | 2.2 | 6.3 | 7.1 |
| 18 - <40 years | |  |  |  |  |
| 1 | 85.2 | 0 | 3.8 | 4.9 | 6.1 |
| 2 | 0 | 84.4 | 7.4 | 6 | 2.2 |
| 40 - 60 years | |  |  |  |  |
| 1 | 86 | 0 | 4.2 | 3.6 | 6.2 |
| 2 | 0 | 84.9 | 7.8 | 5.7 | 1.6 |
| ≥60 years |  |  |  |  |  |
| 1 | 0 | 75.6 | 14.9 | 6 | 3.5 |
| 2 | 88.2 | 0 | 4 | 2.2 | 5.5 |
| Data is presented as prevalence (%). Classes were obtained from best fit model indices using latent class analysis. | | | | | |

**Supplementary table 6**. Baseline characteristics of fluctuation sensitisation subgroups.

|  | **SPT-/SPT+/SPT-** | **SPT+/SPT-/SPT+** | **p-value** |
| --- | --- | --- | --- |
| Total N (%) | 192 (82.8) | 40 (17.2) |  |
| Males | 93 (48.4) | 25 (62.5) | 0.149 |
| Age | 47.8 ± 18.0 | 45.4 ± 18.9 | 0.472 |
| <18 | 19 (9.9) | 4 (10.0) | 0.471 |
| 18- <40 | 38 (19.8) | 12 (30.0) |  |
| 40- <60 | 83 (43.2) | 13 (32.5) |  |
| ≥60 | 52 (27.1) | 11 (27.5) |  |
| **Body composition** | |  |  |
| Waist-height, ratio | 0.6 ± 0.1 | 0.5 ± 0.1 | 0.314 |
| BMI, kg/m^2^ | 26.6 ± 6.2 | 25.7 ± 5.7 | 0.396 |
| FMI, kg/m^2^ | 9.2 ± 4.0 | 8.2 ± 3.8 | 0.140 |
| LMI, kg/m^2^ | 16.3 ± 2.7 | 16.6 ± 2.8 | 0.535 |
| **Biomarkers** | |  |  |
| hsCRP, mg/dL | 1.9 ± 2.8 | 3.1 ± 8.0 | 0.384 |
| Neutrophils, cells/L | 4.1 ± 1.4 | 4.1 ± 1.5 | 0.888 |
| Eosinophils, cells/µL | 149.3 ± 113.8 | 177.6 ± 153.4 | 0.286 |
| **Medication** | |  |  |
| Use of any allergy medication | 2 (1.0) | 3 (7.5) | 0.050 |
| **Lifestyle and early-life events** | | |  |
| Never smokers | 92 (47.9) | 20 (50.0) | 0.919 |
| Former smokers | 64 (33.3) | 12 (30.0) |  |
| Current smokers | 36 (18.8) | 8 (20.0) |  |
| Pack-years | 11.5 ± 18.6 | 11.7 ± 24.9 | 0.948 |
| Passive smoking | 128 (66.7) | 24 (60.0) | 0.533 |
| Urbanicity | 154 (80.2) | 34 (85.0) | 0.630 |
| Residence near main road | 120 (63.2) | 27 (67.5) | 0.735 |
| Exposure to dust | 46 (25.1) | 10 (25.6) | 1.000 |
| Socioeconomic status, score. | 11.9 ± 3.8 | 10.8 ± 4.4 | 0.125 |
| Socioeconomic status, low | 31 (16.1) | 16 (40.0) | 0.001 |
| PM_10_, µg/m^3^ | 21.2 (1.3) | 20.9 (1.1) | 0.107 |
| NO_2_, µg/m^3^ | 20.7 (4.4) | 21.6 (4.1) | 0.283 |
| Parental allergies | 32 (18.1) | 10 (27.8) | 0.270 |
| **Longitudinal analysis- incident conditions/changes in states** | | | |
| New exposures | 55 (30.7) | 13 (34.2) | 0.820 |
| Smoking, quitters | 14 (7.3) | 2 (5.3) | 0.920 |
| Smoking, persistent | 21 (10.9) | 4 (10.3) | 1.000 |
| ∆ BMI, kg/m^2^.year^-1^ | 0.1 ± 0.4 | 0.2 ± 0.3 | 0.375 |
| ∆ FMI, kg/m^2^.year^-1^ | 0.1 ± 0.2 | 0.1 ± 0.2 | 0.301 |
| ∆ Eosinophils, cells/µL.year^-1^ | 0.1 ± 12.6 | 2.4 ± 13.3 | 0.333 |
| ∆ IgE, U/ml.year^-1^ | 4.8 ± 99.0 | -13.4 ± 35.8 | 0.060 |
| Data is presented as mean ± standard deviations or frequency (%). *Significance is considered where p<0.05 for comparisons with the stable non-sensitised group. BMI, body mass index; FMI, fat mass index; LMI, lean mass index; hsCRP, high-sensitivity C-reactive protein; IgE, immunoglobulin E; NO_2_, nitrogen dioxide. ∆ is calculated as (values in Visit 3 minus values in Visit 1)/total visit interval. ∆ IgE is calculated by (values in Visit 3 minus values in Visit 1)/visit intervals between Visits 3 and 2. Significance is considered where p<0.05. | | | |

**Supplementary table 7**. Effects of smoking regressed on longitudinal skin prick test patterns.

|  | **Stable sensitisation** | | **Resolution** | | **New-onset** | | **Fluctuating** | |
| --- | --- | --- | --- | --- | --- | --- | --- | --- |
|  | **OR (95% CI)** | **p-value** | **OR (95% CI)** | **p-value** | **OR (95% CI)** | **p-value** | **OR (95% CI)** | **p-value** |
| **Smoking status** | |  |  |  |  |  |  |  |
| Former smoker (ref. never smoker) | 0.77 (0.61, 0.96) | 0.023 | 0.92 (0.6, 1.41) | 0.701 | 1.06 (0.65, 1.73) | 0.812 | 1.05 (0.66, 1.69) | 0.831 |
| Current smoker (ref. never smoker) | 0.66 (0.50, 0.86) | 0.002 | 0.78 (0.47, 1.3) | 0.338 | 0.60 (0.34, 1.07) | 0.084 | 0.88 (0.50, 1.56) | 0.665 |
| Visit | 1.07 (1.00, 1.15) | 0.036 | 0.97 (0.84, 1.11) | 0.619 | 1.14 (1.00, 1.3) | 0.056 | 1.04 (0.90, 1.2) | 0.620 |
| Former smoker * visit | 1.00 (0.90, 1.11) | 0.944 | 1.02 (0.83, 1.24) | 0.870 | 0.84 (0.67, 1.05) | 0.121 | 0.98 (0.79, 1.21) | 0.842 |
| Current smoker * visit | 0.99 (0.87, 1.12) | 0.826 | 1.11 (0.87, 1.41) | 0.414 | 1.14 (0.87, 1.48) | 0.344 | 1.01 (0.77, 1.32) | 0.968 |
| **Pack years** | |  |  |  |  |  |  |  |
| Pack years | 0.98 (0.97, 0.99) | 0.001 | 0.99 (0.98, 1.01) | 0.546 | 1.00 (0.98, 1.02) | 0.987 | 1.00 (0.99, 1.02) | 0.633 |
| Visit | 1.04 (0.94, 1.16) | 0.435 | 1.00 (0.82, 1.23) | 0.974 | 1.09 (0.88, 1.35) | 0.433 | 1.03 (0.82, 1.28) | 0.809 |
| Pack years * visit | 1.00 (0.99, 1.01) | 0.627 | 1.00 (0.99, 1.01) | 0.840 | 1.00 (0.98, 1.01) | 0.596 | 1.00 (0.99, 1.01) | 0.809 |
| Estimates are odds ratios (OR) with 95% confidence intervals (95% CI) obtained from multinomial logistic regression models adjusted for age groups and sex. Significance is considered where p<0.05. | | | | | | | | |

**Supplementary Table 8**. Polysensitisation across visits in different age strata.

|  | **Stable SPT-** | **Stable SPT+** | **Resolution** | **New-onset** | **Fluctuating** |
| --- | --- | --- | --- | --- | --- |
| **<18 years** |  |  |  |  |  |
| N | 226 | 22 | 11 | 44 | 148 |
| Visit 1 | 0 ± 0 | 2.23 ± 2.33 | 1.09 ± 0.3 | 0 ± 0 | 2.79 ± 2.34 |
| Visit 2 | 0 ± 0 | 2.23 ± 2.33 | 0.73 ± 1.01 | 1.32 ± 1.67 | 3.8 ± 2.21 |
| Visit 3 | 0 ± 0 | 2.23 ± 2.33 | 0 ± 0 | 1.61 ± 1.37 | 3.29 ± 2.35 |
| **18- <40 years** | |  |  |  |  |
| N | 535 | 58 | 65 | 64 | 457 |
| Visit 1 | 0 ± 0 | 2.14 ± 2.11 | 1.58 ± 1.5 | 0 ± 0 | 3.87 ± 2.52 |
| Visit 2 | 0 ± 0 | 2.14 ± 2.11 | 1.15 ± 1.44 | 0.86 ± 1.04 | 4.28 ± 2.62 |
| Visit 3 | 0 ± 0 | 2.14 ± 2.11 | 0 ± 0 | 1.38 ± 0.77 | 3.43 ± 2.34 |
| **40- <60 years** | |  |  |  |  |
| N | 1151 | 108 | 120 | 95 | 684 |
| Visit 1 | 0 ± 0 | 2.15 ± 1.99 | 1.59 ± 1.23 | 0 ± 0 | 3.29 ± 2.46 |
| Visit 2 | 0 ± 0 | 2.15 ± 1.99 | 0.87 ± 1.21 | 0.75 ± 0.98 | 3.70 ± 2.38 |
| Visit 3 | 0 ± 0 | 2.15 ± 1.99 | 0 ± 0 | 1.43 ± 0.88 | 3.11 ± 2.4 |
| **≥60 years** |  |  |  |  |  |
| N | 832 | 37 | 85 | 40 | 264 |
| Visit 1 | 0 ± 0 | 1.46 ± 0.84 | 1.65 ± 1.44 | 0 ± 0 | 2.92 ± 2.45 |
| Visit 2 | 0 ± 0 | 1.46 ± 0.84 | 0.82 ± 1.01 | 0.8 ± 1.14 | 3.42 ± 2.29 |
| Visit 3 | 0 ± 0 | 1.46 ± 0.84 | 0 ± 0 | 1.52 ± 0.96 | 2.64 ± 2.32 |
| Data is presented as frequency or mean ± standard deviation. | | | | | |


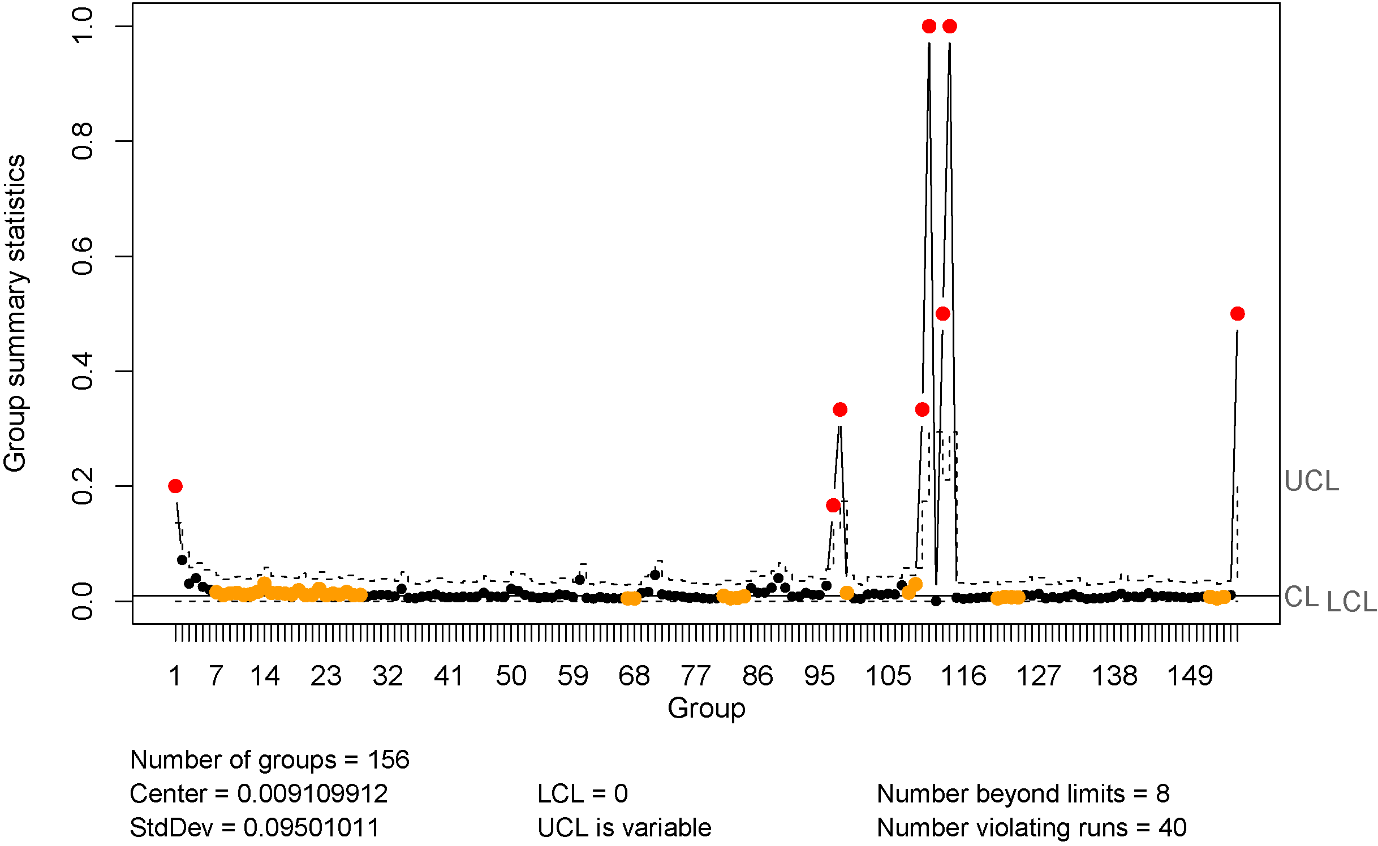


**Supplementary Figure 1**. Control chart (p-chart) of skin prick test control validity for histamine/saline controls across batches and phases for all age strata. *Legend: The central line (CL) represents the average validity rate, while upper/lower control limits (UCL/LCL) indicate binomial variation. Red points denote batches flagged for rule violations such as beyond control limits.*


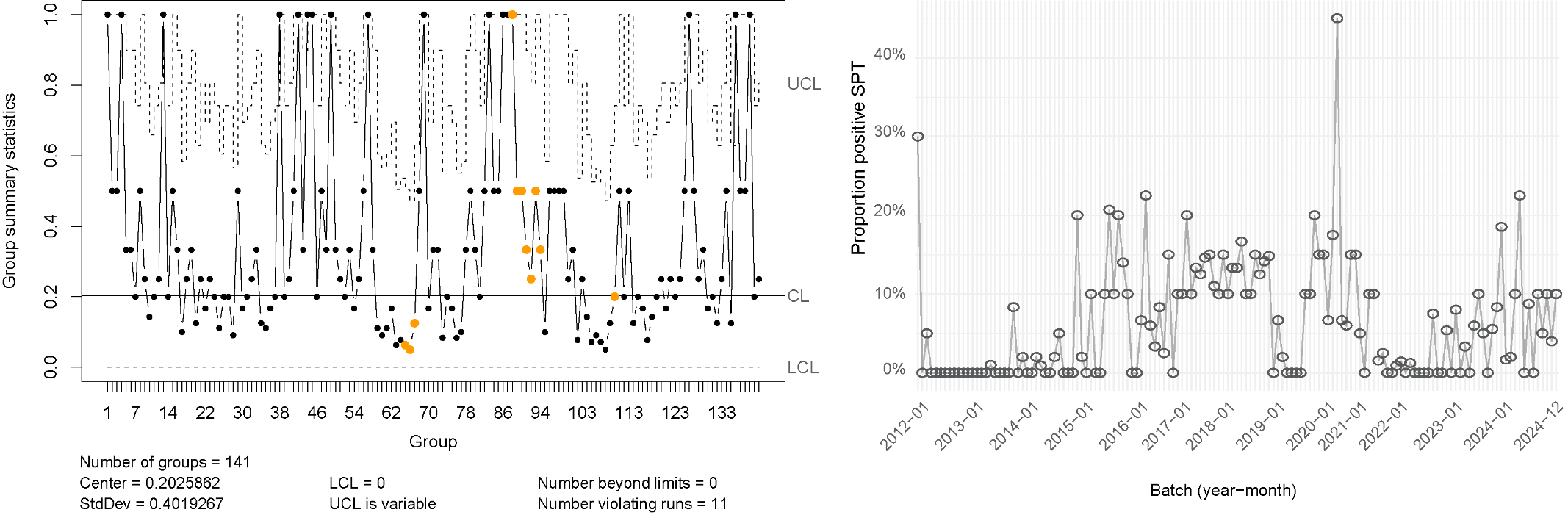


**Supplementary Figure 2**. Control chart (p-chart) of skin prick test control validity for histamine/saline across batches and phases for all age strata in individuals with fluctuating skin prick test across 3 visits. *Legend: The central line (CL) represents the average validity rate, while upper/lower control limits (UCL/LCL) indicate binomial variation. Yellow points denote batches flagged for rule violations such as beyond control limits.*


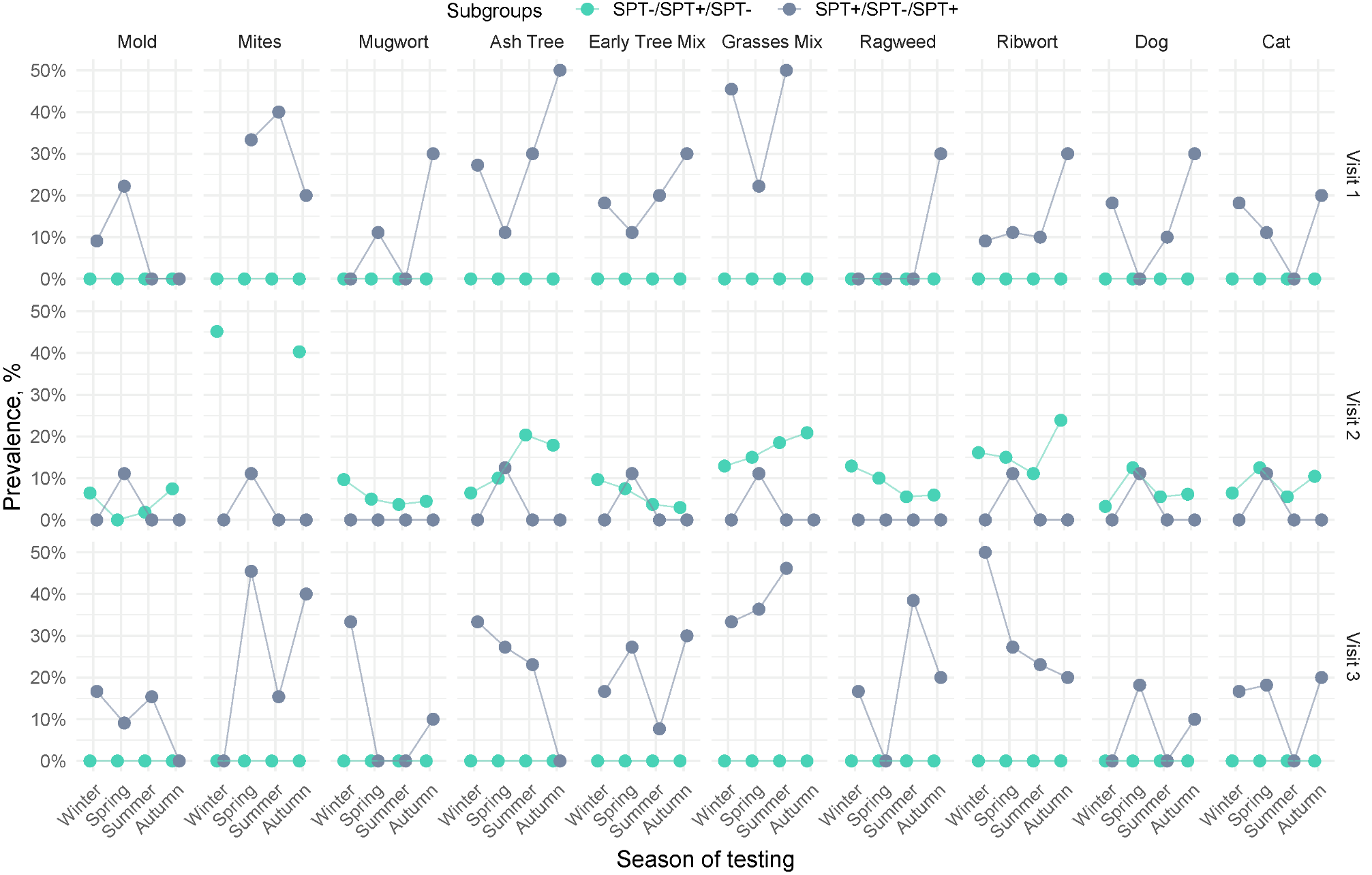


**Supplementary Figure 3**. Seasonal allergen-specific positivity in fluctuation subgroups.
